# PHLDA1 is a shared diagnostic and key mediator of inflammatory fibrosis in heart and kidney

**DOI:** 10.3389/fimmu.2026.1765221

**Published:** 2026-02-05

**Authors:** Lei Hua, Liangru Shen, Yongshou Tao, Chentong Wang, Xiaohang Shao

**Affiliations:** 1 Department of Nephrology and Rheumatology Immunology, Ningbo Medical Centre Lihuili Hospital, Ningbo, Zhejiang, China; 2 Department of Cardiovascular Medicine, The First Affiliated Hospital of Nanjing Medical University, Nanjing, Jiangsu, China; 3 Department of Nephrology, Kang Shen Hospital, the Affiliated Hospital of Jiangsu Food & Pharmaceutical Science College, Suqian, Jiangsu, China; 4 Baicheng County Maternal and Child Healthcare Hospital, Aksu, Xinjiang, China

**Keywords:** fibrosis, heart-kidney, IL-1β, intercellular crosstalk, PHLDA1

## Abstract

**Background:**

Inflammation-driven fibrosis represents a common pathological endpoint in both heart failure (HF) and chronic kidney disease (CKD), which together affect over 1 billion people worldwide. Understanding the shared molecular mechanisms by which inflammation contributes to the pathogenesis of HF and CKD is crucial for enabling early diagnosis and guiding the development of broad-spectrum therapeutic strategies.

**Methods:**

Utilizing multi-omics technologies and machine learning algorithms, we performed an integrative analysis of HF and CKD samples to uncover shared mechanisms underlying inflammation-induced fibrosis. Furthermore, key regulators identified through bioinformatic analysis were experimentally validated using primary cell co-culture assays, gene knockout approaches, and bulk RNA sequencing.

**Results:**

Single-nucleus RNA sequencing (snRNA-seq) revealed concurrent upregulation of IL-1β and Pleckstrin Homology-Like Domain Family A Member 1 (PHLDA1) in both cardiac M1 macrophages and injured proximal tubular epithelial (PTE) cells. PHLDA1 promotes IL-1β expression and the knockout of PHLDA1 suppressed NF-κB signaling and renal fibrosis. Administration of IL-1β induced PHLDA1 expression in cardiac fibroblasts and renal PDGFRβ^+^ cells, suggesting a positive feedback loop that contributes to fibrosis.

**Conclusions:**

In this study, we identified PHLDA1 as a key driver of fibrosis in both the heart and kidney, acting through IL-1β mediated intercellular crosstalk. These findings indicate PHLDA1 as a potential therapeutic candidate for mitigating fibrosis in cardio-renal syndrome.

## Graphical Abstract

**Figure:**
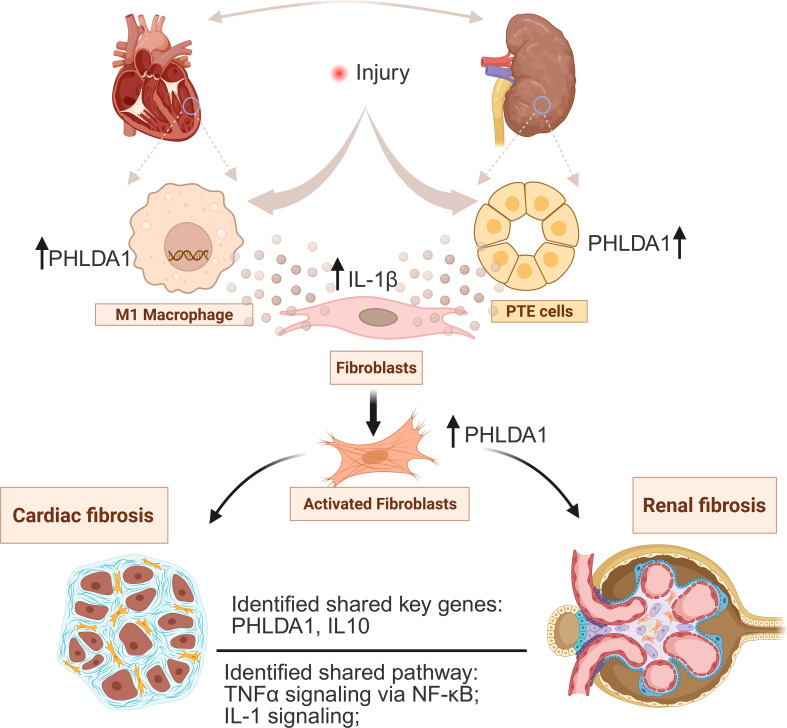

## 1 Introduction

Heart failure (HF) affects over 55 million individuals worldwide and has a five-year survival rate of approximately 50%, even among clinically stable patients (1, 2). Chronic kidney disease (CKD) affects an estimated 850 million people globally (3). While HF and CKD are characterized by impaired cardiac function and the progressive loss of nephron function, respectively, both conditions frequently coexist in individuals due to shared risk factors such as aging, diabetes, and unhealthy lifestyle habits (4). This interconnection has led to the recognition of cardio-renal syndrome (CRS) and cardiovascular-kidney-metabolic syndrome (5).

Fibrosis is considered to be the end-stage of both HF and CKD (6, 7). Fibrosis is a complex and multifaceted pathological process characterized by the excessive accumulation of extracellular matrix (ECM) components within tissues (8). While it plays an indispensable role in tissue homeostasis and organ repair, such as preventing the ventricular rupture after myocardial infarction (MI), excessive activation of this process ultimately disrupts normal tissue architecture and impairs organ function (8). Besides MI-initiated local collagenous scarring, continuous pro-fibrotic stimulation from remote organs also contributes to heart fibrosis. This interstitial fibrosis highlights the mechanism that acute or chronic dysfunction of the heart or kidney leads to similar phenotypes of the other organ (9) (10, 11). Compared to our understanding of heart and kidney diseases as distinct entities, the intertwined pathophysiology and shared etiology of cardiac and renal fibrosis remain poorly understood.

Extensive research has been conducted to clarify the mechanisms involved in fibrosis activation and promotion. So far, inflammation pathways have been considered as activators and promoters with increased significance by mediating cell-cell communications. For instance, interleukin-1β (IL-1β), secreted by M1 macrophages, has been shown to promote cardiac fibrosis by activating cardiac fibroblasts in diabetic mice (12) and genetic disruption of IL-1β alleviates the HF pathogenesis potentially through inhibition of inflammation (13). Meanwhile, in renal fibrosis, injured proximal tubular epithelial (PTE) cells are reported to differentiate into a pro-inflammatory phenotype after kidney injury, promoting the renal mesenchymal cells to secrete ECM, worsening renal function (14). Although progress has been made in understanding fibrosis in each organ individually, the shared inflammatory pathways and cellular crosstalk underlying fibrosis in both the heart and kidney remain incompletely defined.

snRNA-seq has revolutionized our ability to characterize molecular alterations underlying disease, enabling high-resolution analysis at the tissue level and cell-type levels, and even incorporating spatial context. In this study, by leveraging machine learning tools, we performed an integrative analysis of snRNA-seq data from cardiac and renal fibrosis models and identified several diagnostic markers and functional targets implicated in inflammation-driven fibrogenesis. We further employed bulk RNA sequencing to dissect the underlying mechanisms in primary cell cultures. Moreover, our findings highlight PHLDA1 as a central mediator of inflammation-induced fibrosis in both the heart and kidney across multiple cell types. Collectively, these results provide novel insights into the shared fibrotic pathogenesis of these organs and propose potential therapeutic targets to treat tissue fibrosis and preserve organ function in cardio-renal syndrome.

## 2 Materials and methods

### 2.1 Bioinformatic analysis

**Figure 1:**
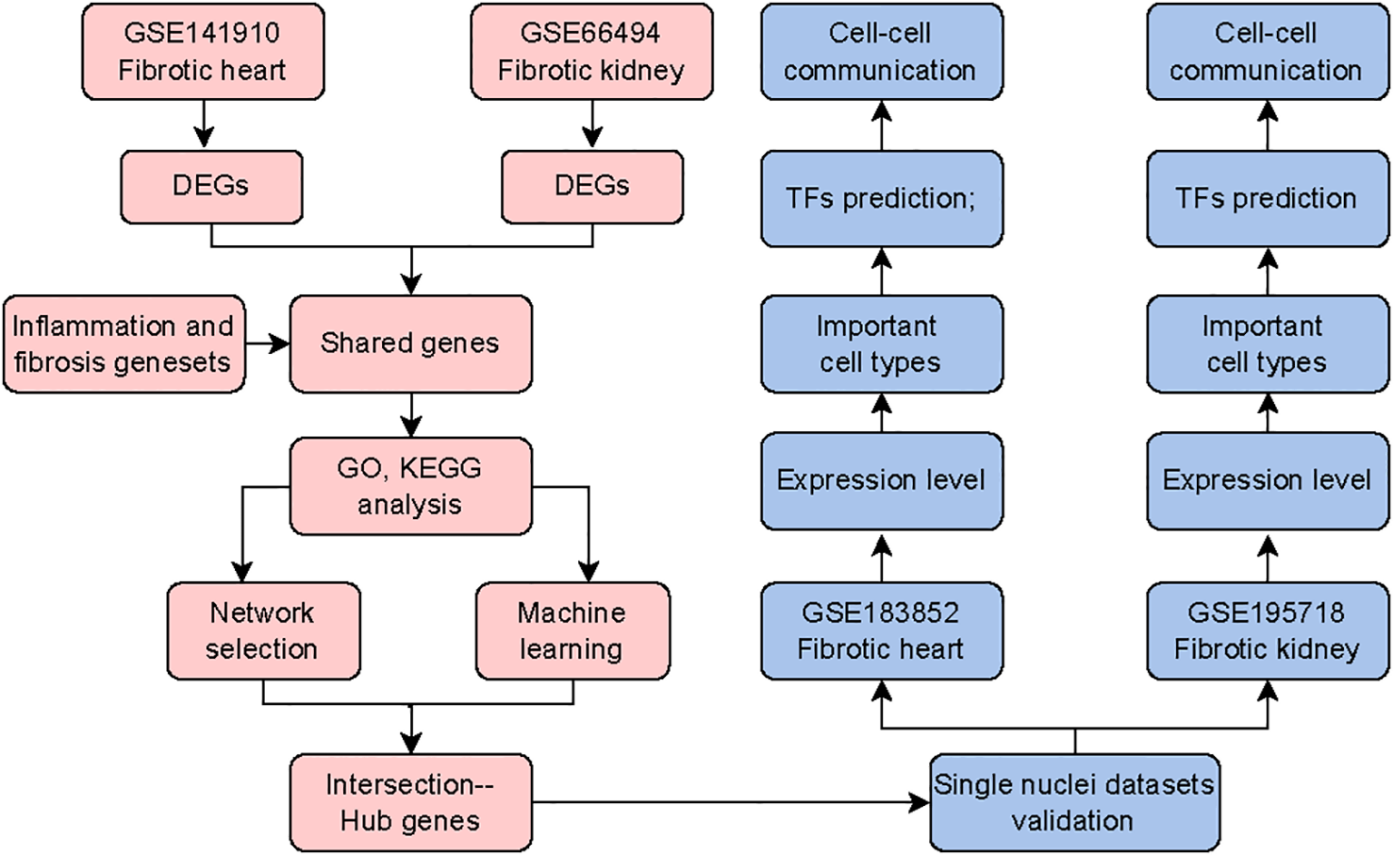
The flow chart of analyzing public datasets.

The analysis flow chart of public data was shown in **Figure 1**.

#### 2.1.1 Preprocessing of RNA-seq data

We analyzed four bulk and single-nuclei RNA-seq datasets for heart and kidney samples from Gene Expression Omnibus (**Table 1**). The first dataset (GSE14910) contains bulk RNA-seq data from 200 left ventricle samples harvested from subjects undergoing transplantation because of end-stage HF and 166 left ventricle control samples from unused donor hearts with normal heart function. The second dataset (GSE66494) contains bulk RNA-seq from 53 renal biopsies with CKD across 8 subtypes, histo-pathologically confirmed the existence of renal fibrosis, and 8 control kidney samples with normal kidney function. The third dataset, GSE183852, studies the differences between end-stage HF and normal control left ventricles. Technically, the GSE183852 dataset contains single-cell RNA sequencing of 7 samples and 38 samples (25 controls and 13 HF) using the snRNA sequencing method. We first filtered out the single-cell RNA sequencing data and kept the snRNA data, since the sample size for snRNA sequencing is larger in GSE183852. The fourth dataset (GSE195718) contains snRNA-seq data from 3 control kidney tissues and 6 fibrotic kidney tissues harvested from kidneys at the end stage of kidney transplantation. For bulk datasets, we normalized the data with the log2 method prior to downstream analysis. For single-cell datasets, we controlled the data quality using the Seurat package (15). Briefly, low-quality cells, defined by having fewer than 300 expressed genes, more than 2,500 expressed genes, or mitochondrial gene expression exceeding 5%, were excluded from the analysis. Detailed information about these datasets is provided in the following:

**Table 1:** Detailed information of the GEO datasets used for key genes identification of the cardiac and renal inflammatory fibrosis.

Accession number	Sample size	Bulk/Single-cell/Single-nuclei	Tissue	Condition
GSE141910	366	Bulk	Left Ventricle	Non-failing VS heart failure
GSE66494	61	Bulk	Renal biopsy	Non-CKD VS CKD
GSE183852	45	Single-cell+ Single nuclei	Left Ventricle	Non-failing VS heart failure
GSE195718	9	Single-nuclei	Renal biopsy	Non-fibrotic VS fibrotic kidney

#### 2.1.2 Gene sets

We downloaded 12,778 genes from the “Harmonizome 3.0” (https://maayanlab.cloud/Harmonizome/) as signature gene sets for inflammation and fibrosis.

#### 2.1.3 Functional enrichment analysis of data from bulk RNA sequencing

To confirm if the inflammation-related and fibrosis-related gene hallmarks (h.all.v2023.1.Hs.symbols.gmt) were activated in HF and CKD, Gene Set Variation Analysis (GSVA) and Gene Set Enrichment Analysis (GSEA) were employed for heart and kidney bulk RNA sequencing data, respectively, using “GSVA” (16) and “clusterProfiler” (17) packages.

Moreover, Gene Ontology (GO) and Kyoto Encyclopedia of Genes and Genomes (KEGG) pathway analysis were performed with the “clusterProfiler” package to discover the main biological processes and pathways for the shared inflammatory fibrosis genes between HF and CKD.

#### 2.1.4 Identification of differentially expressed genes for bulk RNA sequencing

The “Limma” R package was used to identify the differentially expressed genes (DEGs) in the GSE141910 and GSE66494 datasets. DEGs were defined as genes with an adjusted *p*-value < 0.05 and |fold change|≥ 0.75.

#### 2.1.5 Protein-protein interaction construction and key gene selection

The Search Tool for the Retrieval of Interacting Genes/Proteins (STRING) database is a comprehensive database for connecting the known interactions among different genes and proteins. Herein, isolated genes were filtered out of the PPI plot with default parameters. Moreover, the Cytoscape software (version 3.10.3) was used to construct the gene network and choose the key genes with a connection degree number above 5.

#### 2.1.6 Identification and validation of shared diagnostic genes with machine learning

First, the GSE66494 and GSE141910 datasets were merged and divided into the disease group and the control group. We used the “randomForest” R package to classify the disease and control groups. Next, we ranked the genes based on “mean decrease Gini” score and a score higher than 0.25 was considered significant between disease groups (HF and CKD) and the control group. In addition, the LASSO regression method, a more interpretable algorithm compared to Random Forest, was applied to identify significant genes in the merged datasets based on the assumption of a linear relationship between the features and the target. To control overfitting and select the regularization parameter (λ) in the LASSO model, we performed 10-fold cross-validation and chose the λ value that minimized the cross-validation error. Moreover, to assess model stability and ensure reproducibility of feature selection, the analysis was repeated using different random seeds, and the consistency of selected genes across runs was examined.

#### 2.1.7 Single-cell transcriptome analysis and differential expression analysis

After preprocessing the snRNA data, the 2,000 most variable genes were selected using the FindVariableFeatures function to reduce the dimension and identify sub-clusters. Based on the scaled data, Principal Component Analysis (PCA) was performed with variable genes. Next, the RunHarmony function in the “harmony” package was adopted to correct the batch effects across the samples. Subsequently, the FindNeighbors and FindClusters functions were performed on the harmonized data to identify the clusters of the cells. The FindAllMarkers function was used to get the cluster-specific markers. The Wilcoxon’s rank-sum test with the Benjamini-Hochberg method was used to calculate *p*-values and adjusted *p*-values for comparing gene expression among the clusters. Cell types were annotated by mapping marker genes of each cluster to the reference genes from “CellMarkers 2.0” database (18) supplemented with literature.

#### 2.1.8 DEGs identification and GSEA in M1 macrophages

The FindAllMarkers function in the “Seurat” R package was used to calculate gene expression differences in M1 macrophages between the HF and control groups. Subsequently, the clusterProfiler R package (V.4.7.1.2) was used to enrich pathways using “h.all.v2023.1.Hs.symbols.gmt” hallmark gene set. Pathways with an adjusted *p*-value of less than 0.05 were considered significant.

#### 2.1.9 Transcription factor regulon analysis

The Python tool “pySCENIC” is a pipeline used for inferring the importance of the transcription factors (TFs) and their target genes in a single-cell dataset with the Wilcoxon rank-sum test. Herein, we adopted it to discover the regulatory network connecting TFs to their genes in M1 and M2 macrophages. The importance of the regulon-gene connection above 1 was considered significant. Besides, the “AUCell” R package was employed to calculate the regulon activity score for each cell with the regulon list from “pySCENIC”. Furthermore, to identify modules with the most significant TF regulatory networks, the “dendextend” R package was used, and the “h” parameter was set to 5. Visualization of the modules based on the regulon activity score was performed using the “ComplexHeatmap” R package [30].

#### 2.1.10 Cell–cell communication analysis

Since inflammatory fibrosis is driven by multiple cell types, the “CellChat” R package (version 2.1.2) was chosen to explore the possible interactions among cell types and compare the interaction differences among groups. Given that inflammation and fibrosis were the primary focus of this study, macrophages, fibroblasts, myo-fibroblasts, and SMCs were included in the analysis of cardiac fibrosis, while PTE cells and fibroblasts were included for renal fibrosis analysis. Due to limited cell numbers, macrophages were excluded from the fibrosis analysis. To investigate cellular crosstalk, ligand-receptor interactions were predicted using the CellChatDB.human database. Statistically significant interactions (p<0.05) were selected for downstream analysis, and communication networks were constructed and filtered according to the package’s standard tutorial for multiple dataset comparisons, applying all default parameters(https://htmlpreview.github.io/?https://github.com/jinworks/CellChat/blob/master/tutorial/Comparison_analysis_of_multiple_datasets.html).

### 2.2 Experimental validation

#### 2.2.1 Primary cardiac fibroblasts isolation, culture, and stimulation

Detailed primary mouse cardiac fibroblast isolation and culture is as described previously (19). Briefly, hearts from C57BL/6 wild-type male mice were collected, washed, cut into small pieces, and digested with Collagenase I (05172969103, Roche). Then the suspension of the digested mixture was centrifuged, and pellets were seeded in high-glucose DMEM (11965092, Gibco) with 10% fetal bovine serum (15140122, Gibco), 1% streptomycin (100 μg/mL), and penicillin (100 U/mL) (C0222, Beyotime) and cultured in a humidified incubator (37°C, 5% CO_2_). After culturing for 6 hours, the suspensions were removed, and the cardiac fibroblasts adhered to the flasks. Cardiac fibroblasts were cultured in the aforementioned medium, and passages within 10 were used for formal experiments. For stimulation, cardiac fibroblasts were seeded into 12-well plates with a density of 1.5*10^4^/well and cultured overnight. Then replace the medium with 1% FBS, starving cells for 24 hours. For treatment, 100 ng/ml of IL-1β (200-10B, Peprotech) and 15 ng/ml TGF-β (100-21-2UG, Peprotech) were used to stimulate for 24 hours, respectively.

#### 2.2.2 Peripheral blood mononuclear cells and monocytes isolation

First, 9 ml blood was withdrawn from healthy male participants at room temperature. Next, lymphocyte separation medium (TBDTM-0050, TBD Science) was used for PBMC isolation, following the standard operating procedure. In short, 5 ml separation medium was mixed with 4 ml blood and centrifuged for 30 minutes at a speed of 600g. Isolate the PBMC layer for washing, then culturing or freezing. Subsequently, monocytes were isolated from thawed PBMCs using CD14 MicroBeads (130-050-201, Miltenyi Biotec) and a MACS separator (130-092-168, Miltenyi Biotec). Briefly, cells were incubated with CD14 MicroBeads in cold buffer (PBS/2 mM EDTA/0.5% BSA), washed, and passed through an LS column in a MACS separator. Unbound cells were removed with buffer washes, and CD14^+^ monocytes were eluted by plunger flushing.

#### 2.2.3 M1 and M2 macrophages differentiation

Isolated monocytes were counted and seeded in a 24-well plate with a cell density of 4 * 10^5^/well in RPMI 1640 mixed with 10% FBS. Besides, human M-CSF (130-096-491, Miltenyi Biotec) (375 ng/mL) was supplemented to maintain macrophage status. For M1 polarization, cells were stimulated with 10 ng/mL lipopolysaccharide (L4391, Sigma) and 10 ng/mL interferon-γ (300-02; PeproTech) for 6 hours. Regarding M2 polarization, cells were treated with 20 ng/mL interleukin-4 (200-04; PeproTech) and 20 ng/mL interleukin-10 (200-10; PeproTech) for 6 hours.

#### 2.2.4 Ethics and human kidney tissue collection

The present study was approved by the local ethics committee of Ningbo Medical Centre Lihuili Hospital (KY2025SL157-01). Unaffected healthy kidney tissues were used from patients undergoing nephrectomy due to kidney cancer (distant from any cancer as confirmed by a blinded pathologist). Written informed consents were obtained from all patients according to the Declaration of Helsinki.

#### 2.2.5 Generation of human CD10+ PTE and PDGFRβ+ cells

As reported previously (20, 21), CD10 is used for sorting PTE cells, and PDGFRβ^+^ cells are considered mesenchymal cells, acting as the main cellular source of fibrosis in the kidneys. Thus, to validate the results from snRNA data analysis, CD10^+^ and PDGFRβ^+^ were sorted based on the previous experience of our group (21). To ensure renal cells with fibrotic susceptibility, kidney tissues from individuals with long-term CKD (22) were adopted.

Briefly, kidney tissue from the donor (64 years old, male, estimated Glomerular Filtration Rate (eGFR): 34 ml/min/1.73 m^2^) was split into 0.5–1 mm^3^ pieces, and then the gentleMACS system (Miltenyi Biotec, Bergisch Gladbach, Germany) was used for preparing single-cell suspensions following recommended procedures. To sort the PTE cells, cells were pre-treated with Fc-block (TruStain anti-human, BioLegend, dilution: 1:50) for 30 minutes and subsequently incubated with CD10 (HI10a, BioLegend, dilution: 1:40), CD45 (HI30, BioLegend, dilution: 1:40), CD31 (WM59, BioLegend, dilution: 1:40), and CD235a (HI264, BioLegend, dilution: 1:50). Finally, CD10^+^/CD235a^-^/CD31^-^/CD45^-^/DAPI^-^ cell subset was sorted using SONY SH800 cytometer (**Supplementary Figure S1**). PDGFRβ^+^ cell population from another donor (73 years old, male, eGFR: 42 ml/min/1.73 m^2^) was obtained with PDGFRβ antibody (MAB1263, R&D Systems, dilution: 1:100) and separated by magnetic microbeads (130-047-102, Miltenyi Biotec). Isolated renal PTE and mesenchymal cells were cultured in DMEM/F12 GlutaMAX™ medium (10565018, Gibco) supplemented with 10% fetal calf serum (A01125499, Gibco) and 1% streptomycin (100 μg/mL), and penicillin (100 U/mL) (C0222, Beyotime) in a humidified incubator (37 °C, 5% CO2). For subsequent experiments, both cell types were immortalized through lentiviral transduction of SV40LT (E5510S, New England Biolabs) and following our previous protocol (21). Immortalized cell types were cultured as mentioned above.

#### 2.2.6 PHLDA1 knock-out in CD10+ PTE cells

To generate a PHLDA1 KO in the human PTE line, a two-step lentiviral CRISPR/Cas9 system was used. LentiCas9-eGFP (63592, Addgene) was first transduced into cells. Then, the cells that expressed Cas9 were sorted according to eGFP fluorescence (lentiCas9-eGFP) strength. Afterwards, cells were transduced with lentiviral vectors encoding single-guide RNAs (sgRNAs) targeting PHLDA1: pLKO5.sgRNA.EFS.tRFP (57823, Addgene) backbone for sgRNA expression pLKO-PHLDA1-KO-tRFP targeting exon 1 or a non-targeting control sgRNA (pLKO-CRISPR-NT). All constructs expressed turboRFP (tRFP) to enable fluorescent selection of sgRNA-positive cells. Cells were collected based on tRFP signal. Genome editing and transfecting efficiency were confirmed by Sanger sequencing and Flow cytometry analysis (**Supplementary Figure S2**). sgRNA and non-targeting control sequence were provided in **Supplementary Excel 1**.

#### 2.2.7 Establishing the PTE Cell injury model

To induce cellular injury and evaluate transcriptional changes in PHLDA1-knockout and non-targeting control (NT) cells, cultures were exposed to 5 Gy X-ray irradiation for 10 minutes, three times per day, over a period of 3 days.

#### 2.2.8 PTE-PDGFRβ+ kidney cells crosstalk

PDGFRβ^+^ cells were co-cultured for 3 days with day-3 PHLDA1-KO CD10^+^ PTE cells or PHLDA1-NT CD10^+^ PTE cells that had undergone X-ray exposure. Co-culture was performed using 0.4 μm transwells (TCS016006, JET, China). Following co-culture, cells were collected for analysis or prepared for staining.

#### 2.2.9 RNA isolation and quantitative real-time PCR

Total RNA was isolated from cell lines using the RNA isolation kit (1051, Zymo Research) following the manufacturer’s protocol. 300ng RNA was used for the 200 µl cDNA synthesis. Moreover, real-time PCR was performed using SYBR Green Master Mix kit (A25778, Fisher Scientific), and results were calculated with the 2^^(-ΔΔCt)^ method. The primer sequences of all mentioned primers, in this study were provided in **Supplementary Table S1**.

#### 2.2.10 RNA bulk sequencing and downstream analysis

Total RNA from NT and PHLDA1-KO PTE cells was isolated as mentioned above. For bulk mRNA sequencing (RNA-seq), 200 ng total RNA was used for library construction and sequencing was performed by Novogene (Cambridge, UK). Libraries were sequenced on Illumina NovaSeq 6000 flow cells (paired-end, 150bp). Provided FASTQ files were aligned using STAR; reads were counted using featureCounts with -p -t exon -O -g gene_id -s 0. Sequencing quality was assessed using FASTQC. DEGs were analysed with the “DESeq2” R package (23) and genes with adjusted p-value<0.05 and log2|fold change|≥ 1 were considered significant. Downstream settings for GO, KEGG, GSEA, and GSVA analysis were as described above.

#### 2.2.11 Protein isolation and western blot

Regarding protein isolation, cells were lysed in ice-cold RIPA buffer (89900, Thermo Scientific) (with protease/phosphatase inhibitors), incubated (30 min, 4°C), and centrifuged (12,000 rpm, 10 min). Protein concentration was determined via Bio-Rad DC™ Protein Assay (5000121, Bio-Rad). As for the western blot, SDS-PAGE gel was used for electrophoresis running, and proteins were transferred via the Turbo system (1704150, Bio-Rad). After transferring, the PVDF membrane was rinsed with TBS-T (Tris-buffered saline with 0.1% Tween-20) and blocked with 5% BSA in TBS-T for 1hour at room temperature. Next, the membrane was incubated with the PHLDA1 antibody (18263-1-AP, Proteintech, dilution: 1:2000), β-Tubulin antibody (2146S, Cell Signaling Technology, dilution: 1:1000), p65 (ab16502, Abcam, dilution: 1:2000), p-p65 (phospho S536) (ab28856, Abcam, dilution: 1:1000), and b-Actin (4967, Cell Signaling Technology, dilution: 1:1000) overnight at 4°C. Finally, blots were detected after incubation with the second antibody for 1 hour at room temperature.

#### 2.2.12 Immunocytochemistry (ICC) staining

Coverslips are sterilized, coated with 1:100 gelatin, and seeded with PDGFRβ+ cells with a density of 2*10^4^/well in 12 12-well plate, which are allowed to attach and grow. After treatment, cells were fixed using 3% paraformaldehyde, permeabilized with 0.1% Triton X-100 for 20 minutes, and blocked in 2% bovine serum, incubated with a primary antibody overnight at 4°C, washed with PBS, and subsequently incubated with the secondary antibody for 2 h. Primary antibodies include: α-SMA (F3777, Sigma, dilution: 1:100), COL1A1 (ab34710, Abcam, dilution: 1:100), PHLDA1 (sc-23866, Santa Cruz, dilution: 1:50), p65 (ab16502, Abcam, dilution: 1:300), VCAM1 (AF643, R&D Systems, dilution: 1: 400). Nikon A1R confocal microscope was used subsequently for taking images.

#### 2.2.13 p65 overexpression in *PHLDA1-*KO CD10^+^ PTE kidney cells

p65 overexpressed plasmids (pcDNA3.1-p65) and the empty vector (pcDNA3.1 -EV) were provided by GenePharma (Shanghai, China). Transfection of pcDNA3.1-EV and pcDNA3.1-p65 was performed by Lipofectamine 3000 (Thermo Fisher, Waltham, MA, USA) according to the manufacturer’s instructions.

#### 2.2.14 RNA *in situ* hybridization on human kidney sections

RNA *in situ* hybridization (ISH) was performed on 4% formalin-fixed, paraffin-embedded tissue sections with the RNAScope Multiplex Detection Kit V2 (323100, ACD) according to the manufacturer’s guidelines. The RNAscope assay utilized the following probes (ACD): Hs-PHLDA1 (593561-C1), Hs-Col1a1 (1094101-C2), and Hs-p65 (490441-C3).

### 2.3 Statistics

For comparisons between two groups, either an unpaired t-test (for normally distributed data) or the Mann–Whitney test (for non-normally distributed data) was applied. For comparisons among more than two groups, one-way ANOVA with the Brown–Forsythe correction or two-way ANOVA was used accordingly. Results with *p-*values less than 0.05 were considered significant throughout the study.

## 3 Results

### 3.1 Deciphering HF and CKD transcriptional signature

**Figure 2:**
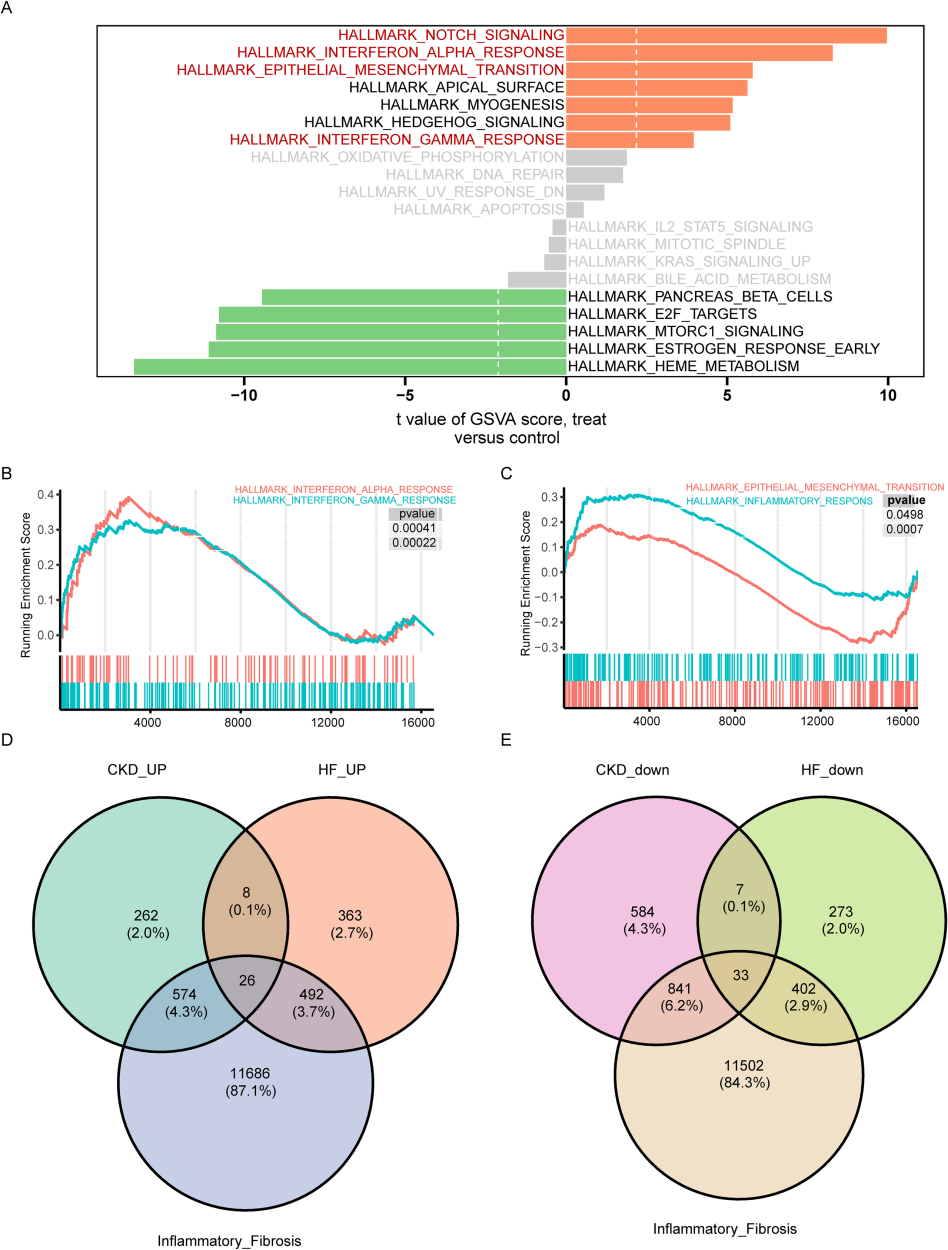
Shared hallmarks and differentially expressed inflammatory fibrosis genes in heart failure (HF) and chronic kidney disease (CKD). **(A)** Gene Set Variation Analysis (GSVA) comparing bulk RNA data between the HF and control groups, highlighting significant hallmark pathways, with upregulated pathways in orange and downregulated pathways in green. **(B, C)** Gene Set Enrichment Analysis (GSEA) of key hallmark pathways between the CKD and control groups. **(D, E)** Venn diagrams illustrating shared upregulated **(D)** and downregulated **(E)** genes intersection with inflammation and fibrosis genesets in both HF and CKD groups.

To understand the overall transcriptional signature of the HF and CKD, GSVA enrichment analysis was performed with the Hallmark Gene Sets. In the HF group, inflammation-related hallmarks, including interferon-α (IFN-α) and interferon-γ (IFN-γ) responses, as well as fibrosis-associated signatures such as Notch signaling and epithelial–mesenchymal transition (EMT), were significantly upregulated (**Figure 2A**). Gene set enrichment analysis (GSEA) of the GSE66494 chronic kidney disease (CKD) dataset revealed significant activation of inflammation and fibrosis-related pathways, suggesting a similar gene expression signature between HF and CKD (**Figures 2B, C**). Specifically, differential expression analysis revealed 889 upregulated and 715 downregulated genes in the HF dataset, and 870 upregulated and 1,465 downregulated genes in the CKD dataset (**Supplementary Figures S3A, B**). By intersecting inflammation and calcification gene sets, we identified 26 upregulated and 33 downregulated inflammatory and fibrosis DEGs shared between the HF and CKD datasets (**Figures 2D, E**).

### 3.2 Shared functional gene enrichment between HF and CKD

To understand the significance and function of shared 59 fibrosis-related DEGs, Gene Ontology (GO) and KEGG analysis were performed. GO analysis revealed enrichment of biological processes related to peptide and protein turnover, including negative regulation of peptidase activity, negative regulation of proteolysis, and response to wounding. Immune responses such as Interleukin-12 (IL-12) production and regulation, and neutrophil chemotaxis were also clustered. Furthermore, apoptosis pathways, particularly those regulating neuronal apoptotic processes, were significantly enriched.

**Figure 3:**
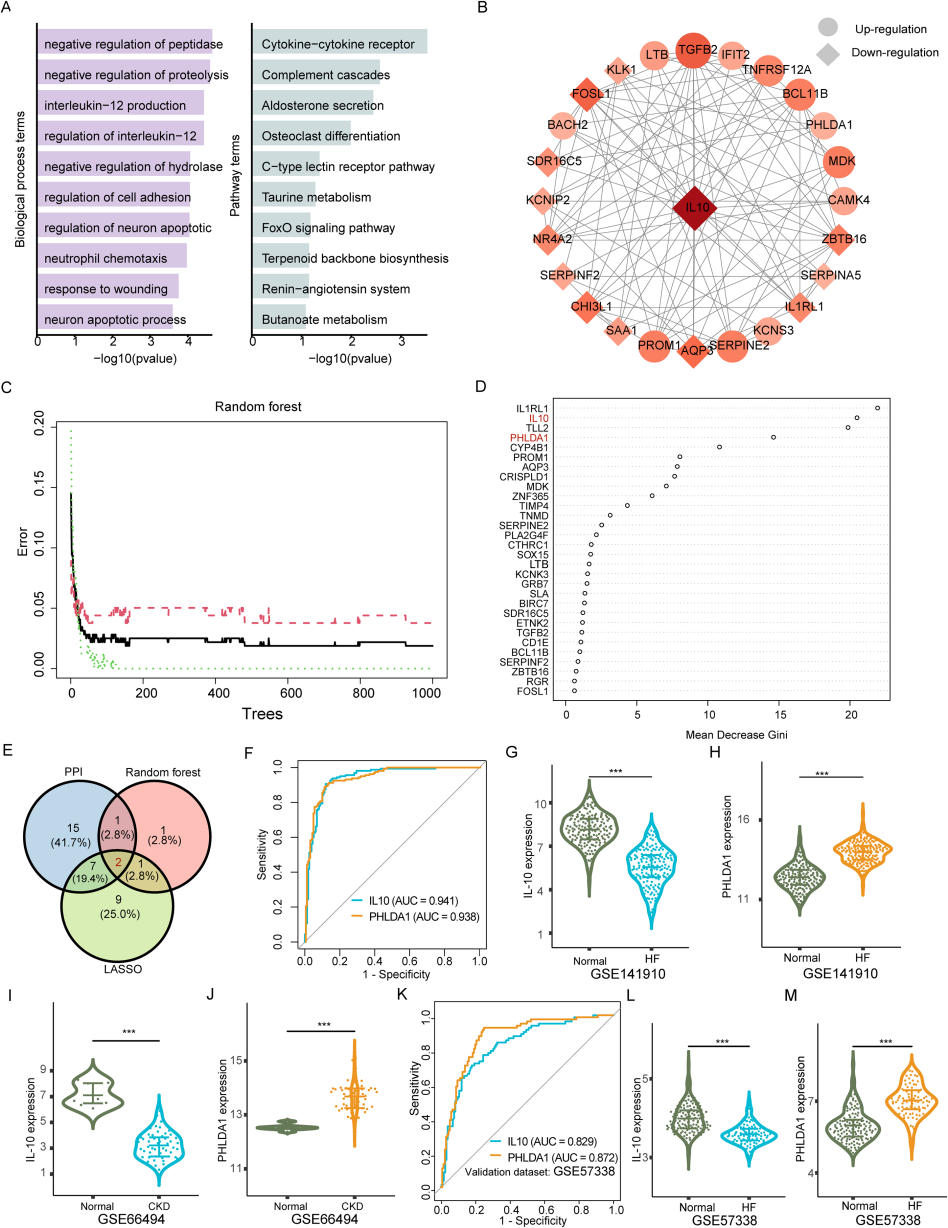
Shared inflammatory fibrosis DEGs function enrichments and key diagnostic genes selection. **(A)**. The top 10 most significant gene ontology terms (left) and Kyoto Encyclopedia of Genes and Genomes pathways (right) are derived from the shared differentially expressed genes in inflammatory fibrosis. The length of the colored bar represents the significance of the term. **(B)**. Protein-protein interaction network based on the key inflammatory fibrosis genes. **(C, D)**. Diagnostic genes selected by the Random Forest algorithm. The error rate of the random forest model relative to the number of trees **(C)** and the ranking of the top genes based on their importance **(D)**. **(E)**. Venn diagram identifying the final candidate hub genes from the intersection of three selection methods: PPI network degree, Random Forest, and Least Absolute Shrinkage and Selection Operator regression. **(F)** Receiver operating characteristic (ROC) curves of IL10 and PHLDA1 for discriminating fibrotic heart failure (HF) samples from normal controls in the discovery HF dataset, with corresponding AUC values. **(G, H)** Violin plots showing normalized expression of IL10 **(G)** and PHLDA1 **(H)** in the HF dataset GSE141910 (Normal vs HF). **(I, J)** Violin plots showing normalized expression of IL10 **(I)** and PHLDA1 **(J)** in the renal dataset GSE66494 (Normal vs CKD). **(K)** ROC curves for external validation of IL10 and PHLDA1 in an independent HF dataset GSE57338, with corresponding AUC values. **(L, M)** Violin plots showing normalized expression of IL10 **(L)** and PHLDA1 **(M)** in the independent HF validation dataset GSE57338 (Normal vs HF). Statistical significance for expression differences was assessed using a two-sided test as described in the Methods; ***p < 0.0001.

KEGG pathway analysis identified several significantly upregulated pathways associated with inflammation and immunity, including the cytokine–cytokine receptor interaction and complement cascade pathways. Additionally, osteoclast differentiation was enriched, suggesting a potential link to tissue remodeling processes (**Figure 3A**). In summary, these results demonstrated that selected genes are closely associated with immune activation (cytokines, complement components, and lectin receptors), as well as fibrosis-promoting signaling pathways related to aldosterone signaling, the renin–angiotensin system (RAS), and FoxO signaling, across both cardiac and renal tissues.

### 3.3 PPI and machine learning algorithms identified key genes in fibrosis

To refine our selection of biologically relevant genes, we constructed Protein-protein interaction networks (PPI). Based on this network, we identified 12 highly connected up-regulated and 13 highly connected down-regulated DEGs for subsequent analyses. (**Figure 3B**). Moreover, to confirm pivotal genes from the merged dataset, we applied a random forest algorithm for feature selection. The out-of-bag (OOB) error decreased sharply with increasing tree numbers and plateaued around 100 trees, indicating robust model convergence and generalization capacity (**Figure 3C**). Feature importance analysis based on the mean decrease in Gini impurity identified five genes, including IL-10, IL-1RL1, Tolloid-like protein 1 (TLL1), PHLDA1, and Cytochrome P450 Family 4 Subfamily B Member 1(CYP4B1), as having high discriminative power, underscoring their potential relevance in the pathogenesis of cardiac and renal fibrosis (**Figure 3D**).

Moreover, the least absolute shrinkage and selection operator (LASSO) regression with 10-fold cross-validation (**Supplementary Figures S3C, D**) was also applied to identify pivotal genes (**Supplementary Table S2**). The optimalλ value was 3.83×10^−4^, giving the minimal cross-validation deviance. To evaluate model stability, the procedure was repeated using multiple random seeds. Selected genes remain consistent across runs, and coefficient estimates for each run are provided in **Supplementary Excel 2**. To enhance robustness, we intersected the results from all selection methods. This integrative approach confirmed that IL-10 and PHLDA1 act as the central genes implicated in the shared inflammation-driven fibrotic processes underlying both HF and CKD (**Figure 3E**). Both IL-10 and PHLDA1 showed strong diagnostic performance, with area under the curve (AUC) and 95% confidence interval (CI) values of 0.941 (95% CI: 0.918-0.964) and 0.931 (95% CI: 0.910-0.952), respectively (**Figure 3F**). Also, the violin plots show the normalized expression of IL10 and PHLDA1 in the HF dataset GSE141910 (**Figures 3G, H**) and the CKD dataset GSE66494 (**Figures 3I, J**). IL10 was downregulated, and PHLDA1 was upregulated in the fibrotic group compared with the normal group. The diagnostic performance of these genes was further validated in an independent HF dataset (GSE57338), yielding an AUC of 0.829 (95% CI: 0.772–0.885) for IL-10 and 0.872 (95% CI: 0.824–0.921) for PHLDA1 (**Figure 3K**) and the violin plot of expression levels of IL-10 and PHLDA1 for GSE57338 was provided in **Figures 3L, M**, showing the significant expression levels for both genes comparing HF to control groups. The GSE104948 validation dataset that contains samples of `ANCA Associated Vasculitis` and normal controls, a kidney disease that further developed into CKD, was used for validation. In this dataset, IL-10 yielded an AUC (95% CI) of 0.626 (0.448–0.805), and PHLDA1 achieved 0.823 (0.687–0.960) (**Supplementary Figure S3E**), and their expression levels were displayed in **Supplementary Figures S3F, G**. Detailed validation dataset information is provided in **Supplementary Table S3**. These results suggest IL-10 and PHLDA1 as biomarkers capable of discriminating between fibrotic and non−fibrotic cardiac and renal tissues. Collectively, IL-10 and PHLDA1 were prioritized as key genes implicated in inflammation-driven fibrosis across organs.

### 3.4 snRNA transcriptome analysis of cardiac and renal fibrosis samples

**Figure 4:**
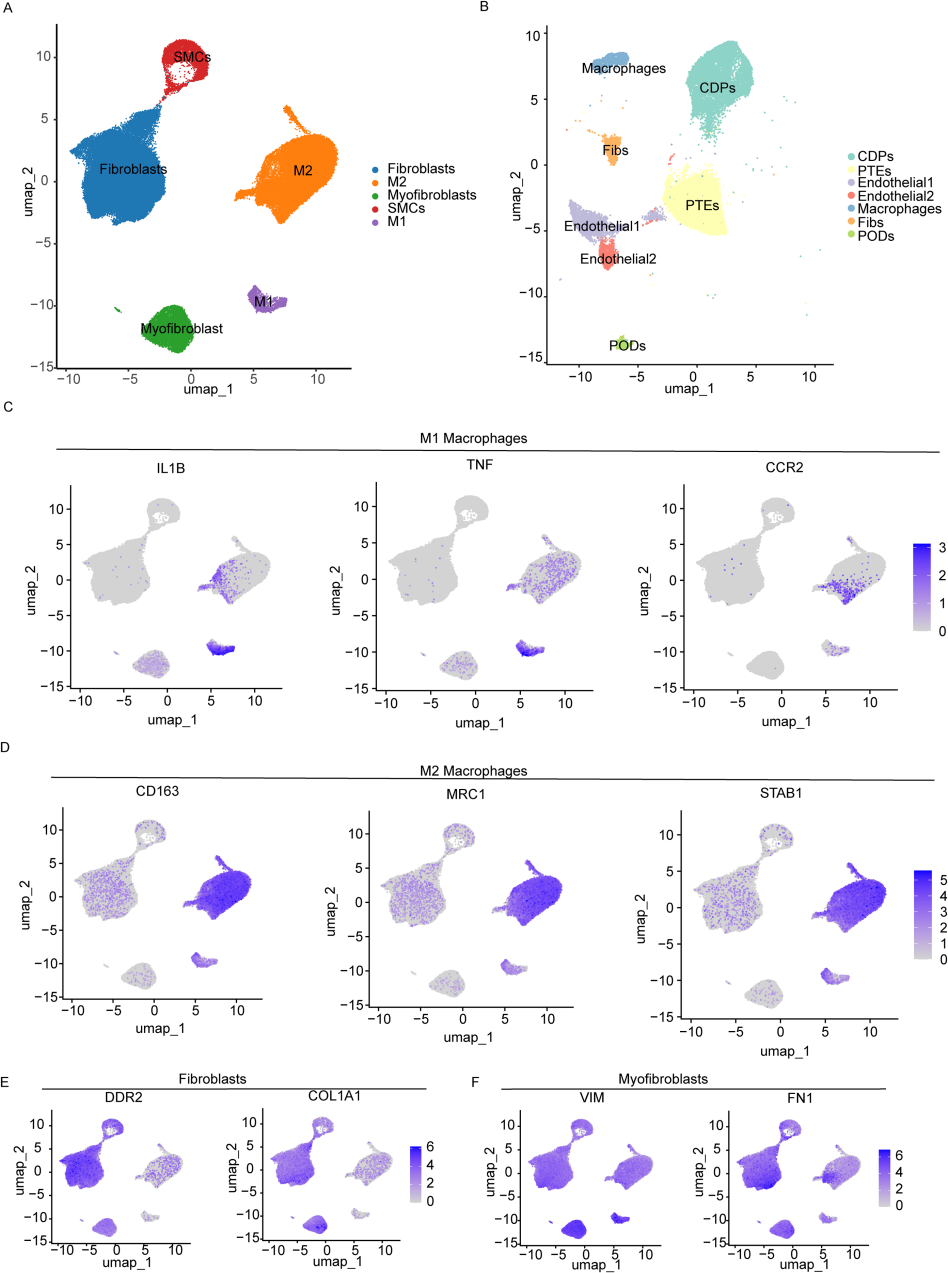
Major cell types during heart and kidney tissue fibrosis. **(A)** Uniform Manifold Approximation and Projection (UMAP) plot visualizing the 5 major cell types identified in the human heart single-nuclei RNA sequencing (snRNA-seq) dataset (GSE183852). **(B)** UMAP plot visualizing the 7 major cell types identified in the human kidney snRNA-seq dataset (GSE195718). **(C–F)** UMAP plots displaying the expression levels of canonical marker genes used to validate cell type annotations. Expression levels are represented by a color gradient from gray to purple, including M1 Macrophages **(C)**, M2 Macrophages **(D)**, Fibroblasts **(E)**, and Myo-fibroblasts **(F)**.

To investigate the cell-type-specific roles of IL-10 and PHLDA1 in cardiac and renal fibrosis, we conducted further analyses using snRNA-seq datasets GSE183852 and GSE195718, corresponding to heart and kidney tissues, respectively (**Figures 4A, B**). Specific cell populations were annotated based on the expression profiles of established cell-type markers (**Figures 4C–F**). Cell-type annotation was defined by canonical gene signatures, including markers for M1 macrophages (IL1B, TNF, CCR2), M2 macrophages (CD163, MRC1, STAB1), fibroblasts (COL1A1, DDR2), and myofibroblasts (VIM, FN1). Smooth muscle cells were identified by the expression of TAGLN, ACTA2, and MYH11. For the renal snRNA-seq dataset GSE195718, endothelial cells were annotated using PECAM1 and CD34 (**Supplementary Figure S4B**); macrophages were marked by CD68, C1QA, and CD14 (**Supplementary Figure S4C**); fibroblasts were defined by PDGFRB, COL1A1, and ACTA2 (**Supplementary Figure S4D**); PTEs were annotated with LRP2, ALDOB, and CUBN (**Supplementary Figure S4E**).

### 3.5 PHLDA1 contributed to cardiac fibrosis, potentially through crosstalk between M1 macrophages and cardiac fibroblasts

**Figure 5:**
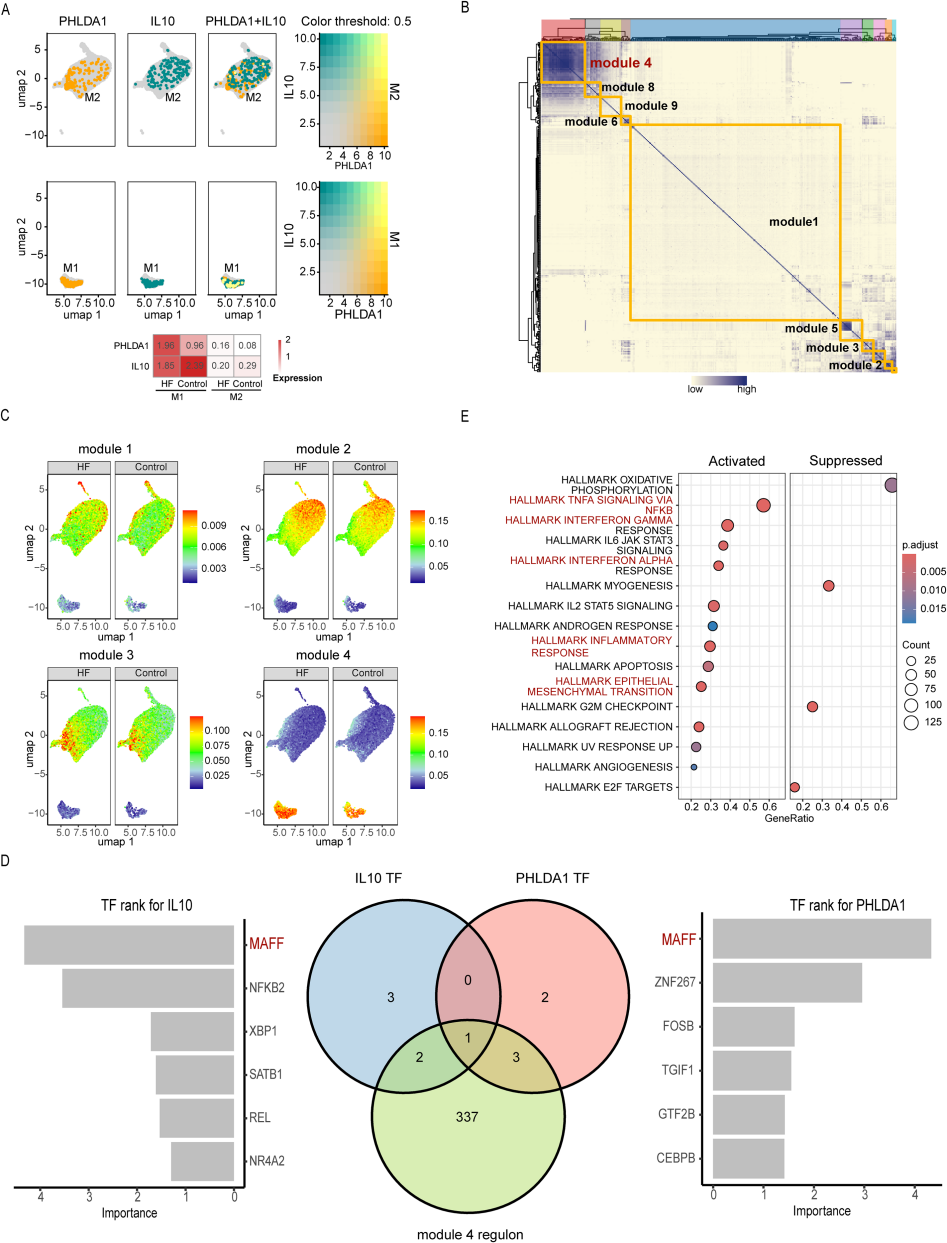
IL-10 and PHLDA1 expression patterns and key regulon network selection in macrophages during cardiac fibrosis. **(A)** Feature plots illustrating the expression and co-expression of IL-10 and PHLDA1 in M1 and M2 macrophages (dataset GSE183852) and the heatmap showing the average expression of IL-10 and PHLDA1 in M1 and M2 macrophages, comparing heart failure (HF) and control groups. **(B)** Heatmap displaying the 10 transcription factors (TFs) modules in macrophages. **(C)** Uniform Manifold Approximation and Projection (UMAP) plot visualizing the enrichment of the Top 4 TF modules in M1 and M2 macrophages between HF and control groups. **(D)** Bar plots demonstrating the TF rank of IL-10 (Left) and PHLDA1 (Right) from the regulatory network of M1 macrophage, and the Venn diagram (middle) showing their important TFs intersection with TFs in module 4. **(E)** Gene Set Enrichment Analysis (GSEA) of Hallmark Pathways in M1 Macrophages. The bubble plot depicts hallmark signaling pathways that are significantly dysregulated in M1 macrophages HF group compared to control samples. The pathways are categorized into two major groups: “Activated” (right panel) and “Suppressed” (left panel) in the HF condition.

Initial gene expression analysis revealed that IL-10 and PHLDA1 were highly expressed in both M1 and M2 macrophage populations, with particularly elevated levels in M1 macrophages. Moreover, this expression pattern in HF was consistent with findings from bulk RNA sequencing (**Figure 5A**).

**Figure 6:**
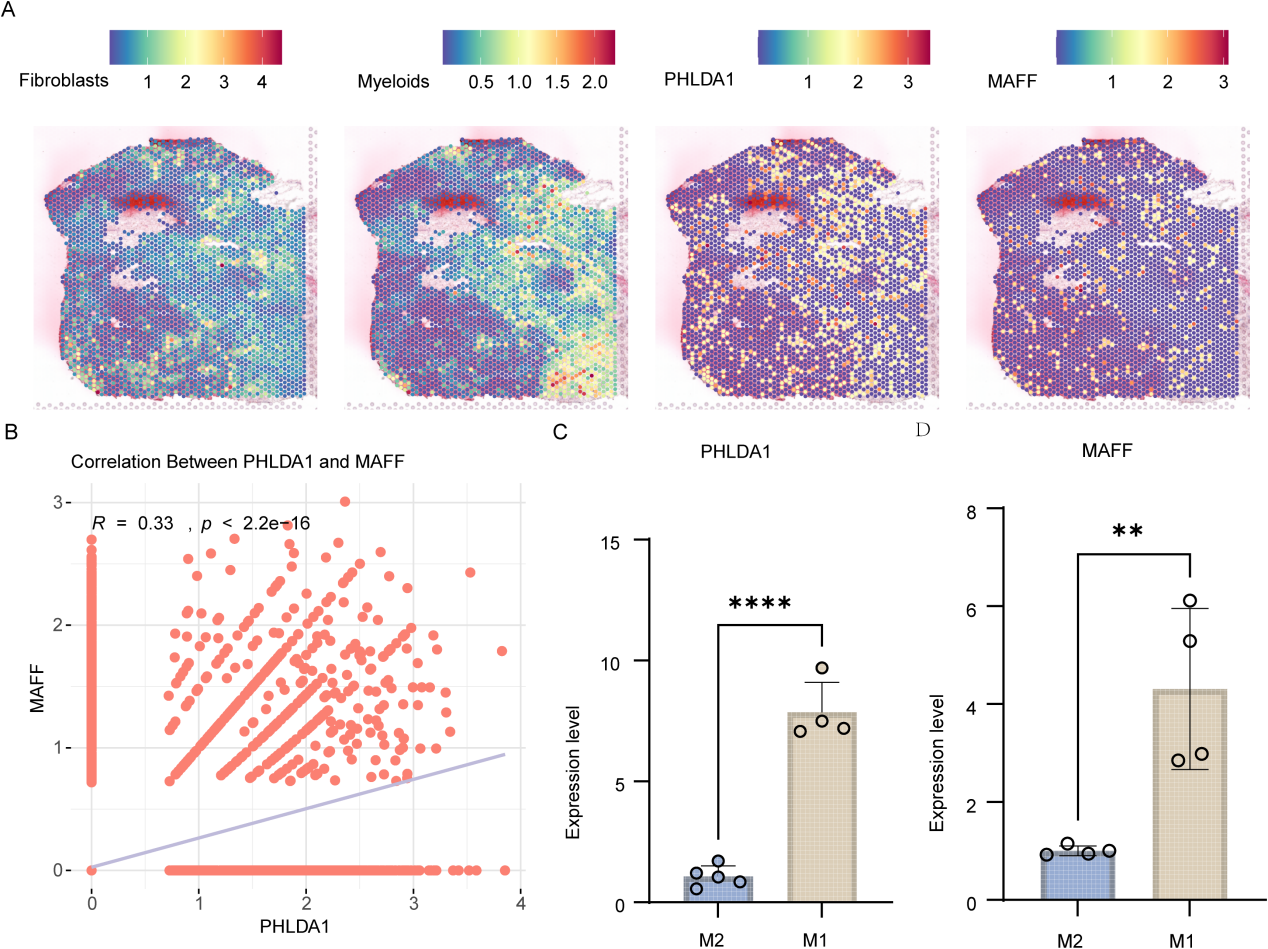
PHLDA1 and MAFF were significantly up-regulated in M1 macrophages during cardiac fibrosis. **(A)** Spatial transcriptomics maps of a representative tissue section showing the distribution of fibroblasts (first panel), myeloid cells (second panel), and the expression patterns of PHLDA1(third panel) and MAFF (fourth panel). Their expression levels are represented by the color scale. **(B)** Scatter plot demonstrating the correlation between the expression levels of PHLDA1and MAFF in GSE183852. **(C, D)** q-PCR detecting the gene expression levels of PHLDA1 **(C)** and MAFF **(D)** in M2 and M1 macrophages differentiated from peripheral blood mononuclear cells. **(F)** q-PCR evaluating Phlda1 expression levels in IL-1β or TGF-β stimulated cardiac fibroblasts, gene expression was normalized to GAPDH. Data are presented with mean ± SD; ordinary one-way ANOVA was performed in F; *p < 0.05. q-PCR, Quantitative Polymerase Chain Reaction.

Given that IL-10 and PHLDA1 were enriched in macrophages, we focused on M1 and M2 macrophage subsets to investigate the upstream regulatory networks. Using regulon activity scores derived from the pySCENIC, we clustered the transcriptional regulatory network into ten distinct modules (**Figure 5B**). Modules 1–3 were primarily enriched in M2 macrophages, whereas module 4 showed predominant activation in M1 macrophages (**Figure 5C**). Notably, module 4 exhibited higher activity in HF samples compared to controls, prompting its selection for further analysis. Transcription factors (TFs) regulating IL-10 and PHLDA1 were extracted from the module 4 regulon and ranked by importance (**Figure 5D**). Intersection analysis identified MAFF as a key TF within M1 macrophages that co-regulates both IL-10 and PHLDA1 in the module 4 network. Gene set enrichment analysis (GSEA) of M1 macrophages further revealed significant activation of inflammatory pathways, including TNFα signaling via NF-κB, Interferon-γ and Interferon-α responses, IL6–JAK–STAT3 signaling, and general inflammatory response, as well as remodeling-associated pathways such as epithelial–mesenchymal transition (**Figure 5E**). Re-analysis of previously published spatial transcriptomic data from fibrotic heart tissue demonstrated that PHLDA1 and MAFF were co-expressed in regions enriched for fibroblasts and myeloid cells (**Figure 6A**). Moreover, Spearman correlation analysis confirmed a significant positive correlation between PHLDA1 and MAFF expression (*R* = 0.33, *p* < 0.001) (**Figure 6B**). Furthermore, PBMC-induced M1 and M2 were used for experimental validation. As shown in **Figure 6C**, PHLDA1 and MAFF gene expression in M1 were significantly higher than in M2. These data suggest that the MAFF-PHLDA1-inflammation pathway contributes to cardiac and renal tissue fibrosis via cytokine-mediated intercellular communication.

**Figure 7:**
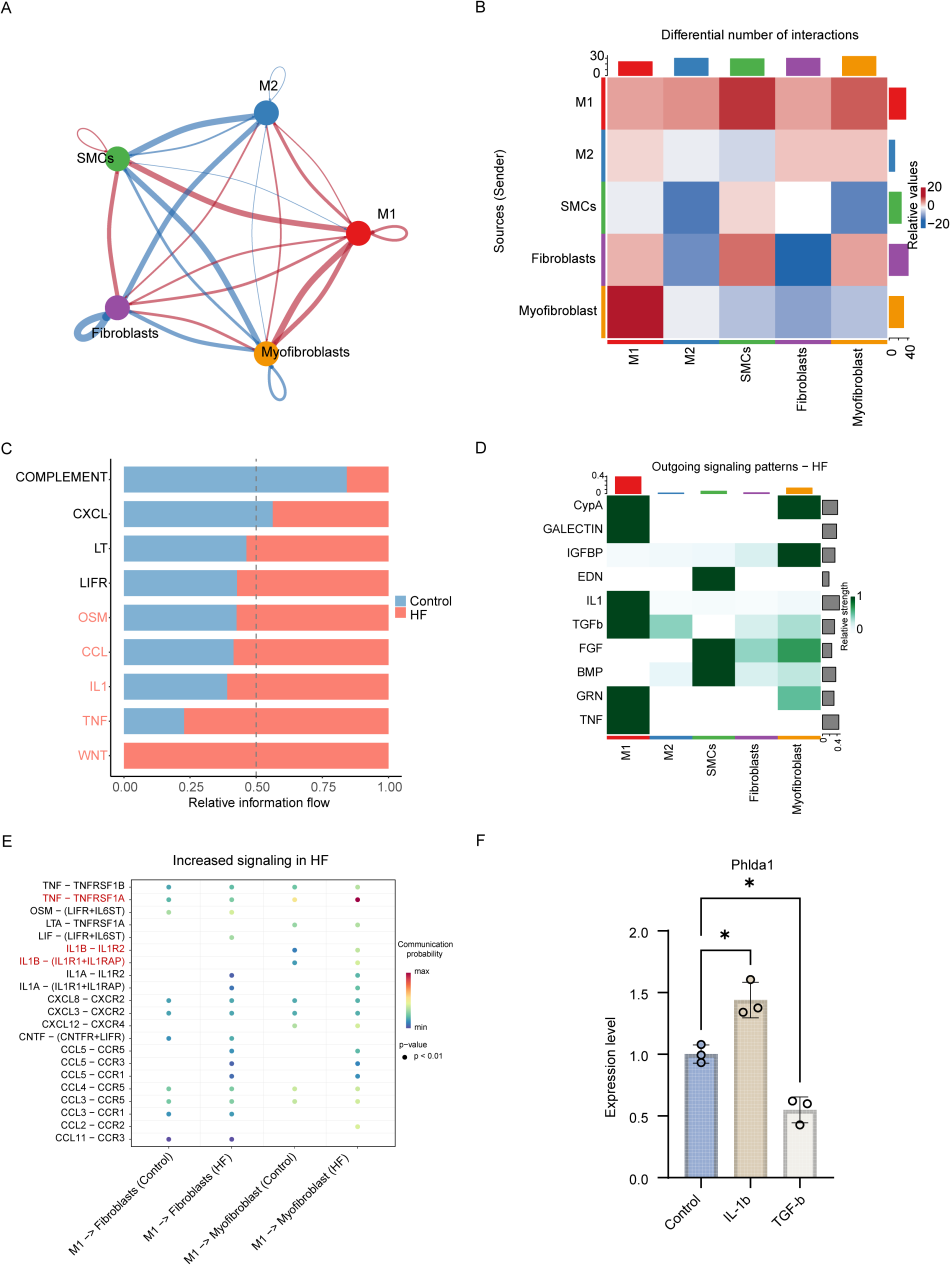
Cell communication analysis comparing heart failure (HF) and control groups reveals a significant rewiring of the intercellular signaling network in the cardiac microenvironment. **(A)** The overall communication network illustrating interactions between major cell types, including macrophages (M1, M2), fibroblasts, myofibroblasts, and smooth muscle cells (SMCs). Edge thickness and color representing communication strength and direction. **(B)** Differential interaction heatmap showing changes in the number of inferred interactions between sender (source) and receiver (target) cell types in HF compared with control. **(C)** Global information flow analysis showing a systemic increase in signaling strength in HF (red) compared to control (blue) across the key pathways. **(D)** Heatmap of outgoing signaling patterns in HF, showing pathway activity enriched across sender cell types. **(E)** Bubble plot of representative ligand–receptor pairs with increased communication probability in HF, highlighting enhanced signaling from M1 macrophages to fibroblasts and myofibroblasts. Dot color reflects communication probability (low to high), and dot size indicates statistical significance. **(F)** q-PCR evaluating Phlda1 expression levels in IL-1β or TGF-β stimulated cardiac fibroblasts, gene expression was normalized to *Gapdh*. Data are presented with mean ± SD; unpaired Student t-tests were performed in B, I, and J; *p < 0.05. HF, heart failure; M1, M1 macrophage; M2, M2 macrophage; SMCs, smooth muscle cells; LT, lymphotoxin; LIFR, leukemia inhibitory factor receptor; OSM, oncostatin M; CCL, C–C motif chemokine ligand; IL-1, interleukin-1; TNF, tumor necrosis factor; WNT, wingless-type signaling pathway; CypA, cyclophilin A; GALECTIN, galectin; IGFBP, insulin-like growth factor binding protein; EDN, endothelin; TGF-β (TGFB), transforming growth factor beta; FGF, fibroblast growth factor; BMP, bone morphogenetic protein; GRN, granulin; CNTF, ciliary neurotrophic factor; LIF, leukemia inhibitory factor; OSMR, oncostatin M receptor; LTA, lymphotoxin alpha; CXCL, C–X–C motif chemokine ligand; IL-1β, interleukin-1 beta; IL1R, interleukin-1 receptor; IL1RAP, interleukin-1 receptor accessory protein; IL1RL1, interleukin-1 receptor-like 1; CXCR, C–X–C chemokine receptor; CCR, C–C chemokine receptor; RT–qPCR, reverse transcription quantitative polymerase chain reaction; Phlda1, pleckstrin homology-like domain family A member 1.

To elucidate the intercellular communication underlying inflammation and fibrosis in HF, we employed the CellChat tool to analyze cell–cell interactions among key effector populations, including macrophages, smooth muscle cells, fibroblasts, and myofibroblasts. Bidirectional interactions between M1 macrophages and myofibroblasts were markedly increased in the HF group (**Figures 7A, B**). Signal information flow analysis further revealed upregulation of TGF-β and IL-1 signaling pathways in HF samples (**Figures 7C, D**). Detailed ligand–receptor pair analysis identified elevated communication probabilities for TNF–TNFRSF1A and IL1B–receptor interactions between M1 macrophages and myofibroblasts in HF (**Figure 7E**). To functionally validate these findings, cardiac fibroblasts were stimulated with 100 ng/mL IL-1β or 15 ng/mL TGF-β for 24 hours. Notably, PHLDA1 expression was upregulated following IL-1β treatment, while it was dramatically downregulated upon TGF-β treatment, suggesting differential regulatory effects of inflammatory versus fibrotic cues on PHLDA1 expression (**Figure 7F**).

### 3.6 PHLDA1 mediated cell-cell communications between PTEs and fibroblasts during renal fibrosis

**Figure 8:**
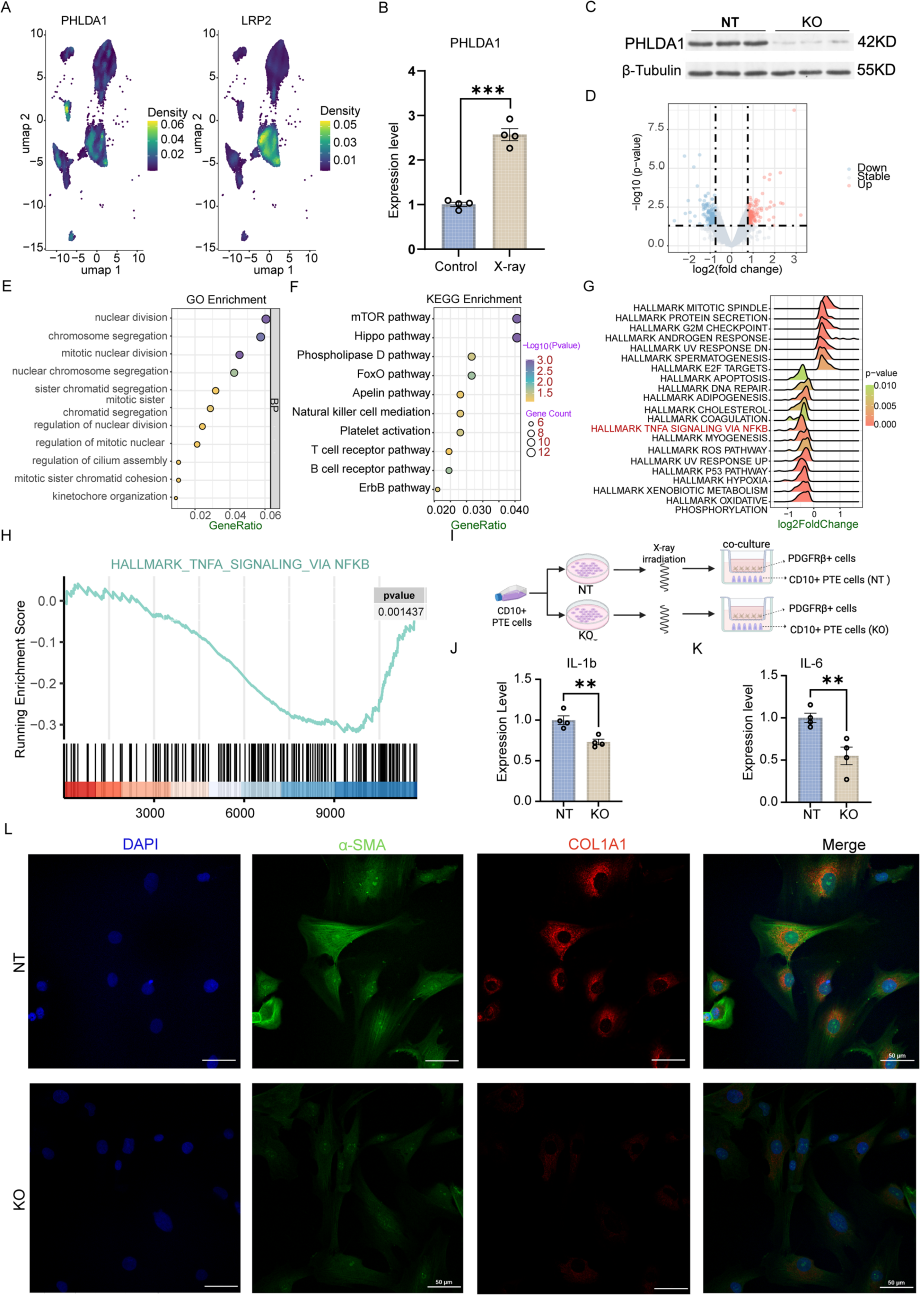
PHLDA1 promotes renal fibrosis by regulating the crosstalk between proximal tubular epithelial (PTE) and PDGFRβ^+^ kidney cells. **(A)** Density plots from single-nuclei RNA sequencing data (GSE195718) showing the co-expression of PHLDA1with the PTE cells in fibrotic kidney. **(B)** PHLDA1 expression in PTE cells following X-ray-induced injury compared to control. **(C)** Western blot validating PHLDA1 knockout (KO) efficiency in PTE cells compared to the Non-Targeting (NT) group. **(D)** Volcano plot of differentially expressed genes (DEGs) between PHLDA1-KO and NT PTE cells after x-ray injury. **(E, F)** Pathway enrichment analyses of differentially expressed genes. Bubble plots show significantly enriched Gene Ontology (Biological Process) terms **(E)** and KEGG pathways **(F)**, ranked by significance (color, -log_10_ (p-value)) and number of genes involved (size). **(G)** Gene Set Variation Analysis (GSVA) of hallmark signalling pathways in PHLDA1-KO versus NT group PTE cells. The density curves illustrate the distribution of pathway activity scores (log2FoldChange) between the two groups. The color gradient from green to red corresponds to the statistical significance. **(H)** Gene Set Enrichment Analysis of TNFα signalling via NF-κB pathway. **(I)** Brief process of PDGFRβ^+^ kidney cells and PTE cells co-culture. **(J, K)** q-PCR validation of IL-1β **(J)** and IL-6 **(K)** expression in NT and PHLDA1-KO PTE cells after x-ray exposure. **(L)** Immunofluorescence staining of fibrosis markers (α-SMA, COL1A1) in PDGFRβ^+^ kidney cells co-culturing with PHLDA1-KO and NT groups, respectively. **(L)** Bar, 50 μm. Data are presented with mean ± SD; unpaired Student t-tests were performed and gene expression was normalized to GAPDH in B, I, and J; **p < 0.01, ***p < 0.001, ****p < 0.0001. q-PCR, Quantitative Polymerase Chain Reaction.

PHLDA1 expression was enriched in PTE cells, indicating its potential role in PETs and tissue pathogenesis (**Figure 8A**). To further investigate the function of PHLDA1 in renal PTE cells, primary PTE cells were isolated from human fibrotic kidney tissues using FACS sorting followed by cellular immortalization. In addition, X-ray irradiation was used to mimic injury in PTE cells in fibrotic kidneys. As shown in **Figure 8B**, PHLDA1 expression was significantly upregulated in response to X-ray–induced injury. To determine whether PHLDA1 plays a causal role in PTE cell injury, PHLDA1 was knocked out. As confirmed by Western blot analysis in **Figure 8C**, PHLDA1 expression was almost abolished in *PHLDA1*-KO PTE cells. RNA bulk sequencing identified 149 upregulated and 211 downregulated genes in KO versus non-targeting (NT) PTE cells (**Figure 8D**). The biological processes associated with these differentially expressed genes were primarily related to nuclear function (**Figure 8E**). KEGG analysis revealed that loss of *PHLDA1* led to significant enrichment of the mTOR, Hippo, phospholipase D, FoxO, apelin, natural killer cell–mediated cytotoxicity, platelet activation, T cell receptor, B cell receptor, and ErbB signaling pathways (**Figure 8F**). GSVA analysis demonstrated that *PHLDA1* knockout significantly suppressed the HALLMARK TNFα signaling via NF-κB, HALLMARK apoptosis, and HALLMARK reactive oxygen species pathways. Consistently, GSEA confirmed that the TNFα signaling via NF-κB hallmark was markedly downregulated in PTE cells lacking PHLDA1 (**Figures 8G, H**). Furthermore, we constructed a co-culture system for CD10+ PTE cells and PDGFRβ^+^ cells, mimicking the inflammation and fibrosis process of the human kidney (**Figure 8I**). During the co-culture, expression of inflammatory markers (IL-1β and IL-6) was significantly reduced in the *PHLDA1* KO group (**Figures 8 J, K**). Consistently, cell staining revealed that the expression levels of α-SMA and COL1A1 in PDGFRβ^+^ cells were also reduced in the group co-cultured with *PHLDA1*-KO CD10^+^ PTE cells (**Figure 8L**), suggesting that *PHLDA1* deletion in CD10^+^ PTE cells could alleviate the renal fibrotic process.

**Figure 9:**
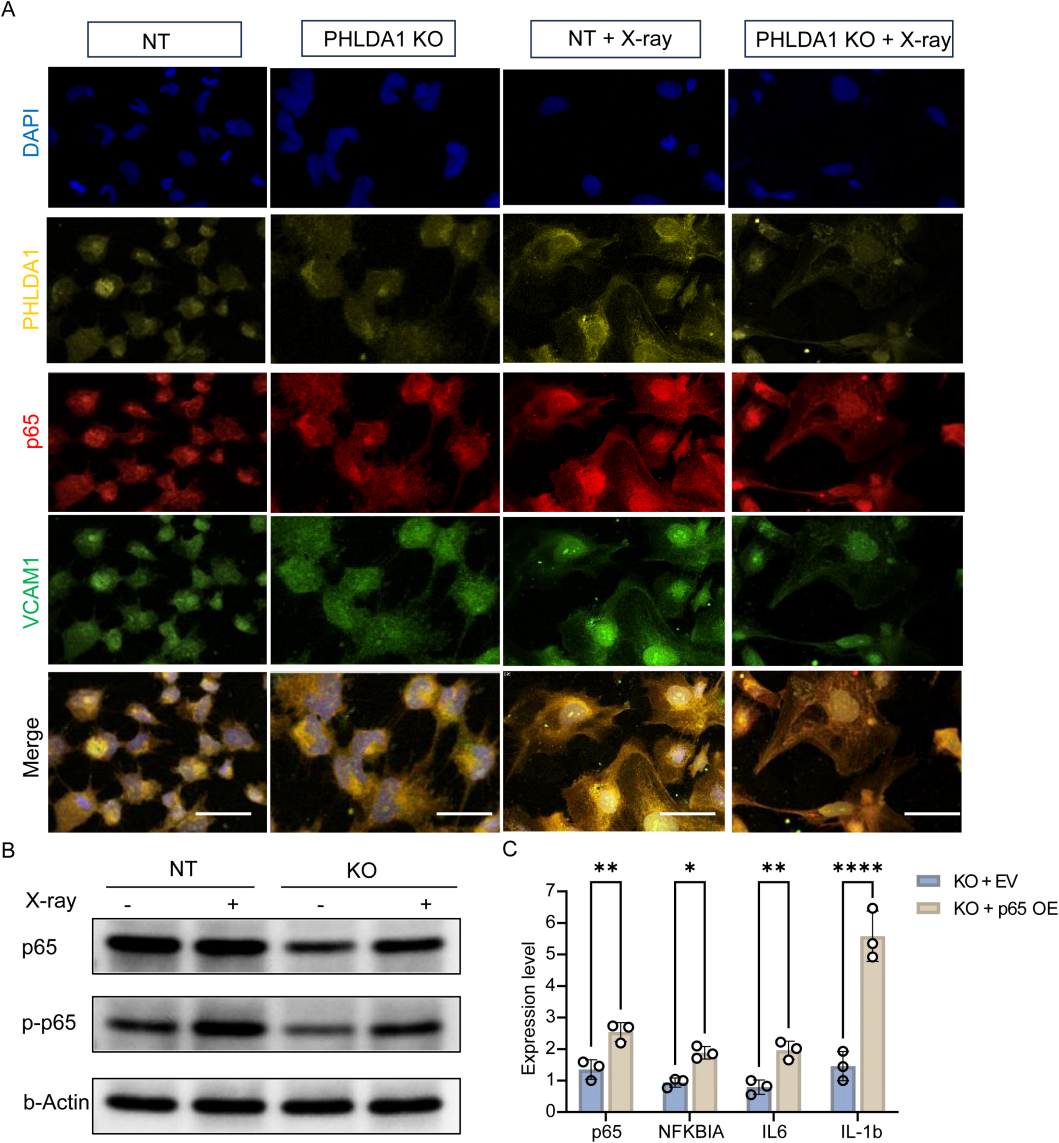
PHLDA1 regulates the inflammation levels of CD10^+^ PTE kidney cells via the NF-κB pathway. **(A)** Representative immunocytochemistry (ICC) images of CD10^+^ PTE cells under the indicated conditions for 3 h. Nuclei were counterstained with DAPI (blue). PHLDA1 (yellow), p65 (red), and VCAM1 (green) are shown with merged images in the bottom row. n = 4 for each group, scale bars, 50 μm. **(B)** Western blot of total p65 and phosphorylated p65 (p-p65) in NT and PHLDA1-KO CD10+ PTE cells with or without X-ray exposure. **(C)** Relative mRNA expression of NF-κB–associated inflammatory markers in PHLDA1-KO CD10+ PTE cells transfected with empty vector (EV) or p65 overexpression plasmid (OE). Gene expression was normalized to GAPDH. Data are presented with mean ± SD; Statistical significance was determined by two-way ANOVA; *p < 0.05, **p < 0.01, ****p < 0.0001.

Furthermore, to validate that PHLDA1 regulates the NF-κB pathway, the expression of p65 and Vascular Cell Adhesion Molecule 1 (VCAM1), key components of the NF-κB signalling cascade, was assayed with ICC staining. The expression of p65 and VCAM1 was significantly reduced in PHLDA1 KO cells after X-ray exposure compared to the control (**Figure 9A**). Concordantly, both total p65 and phosphorylated p65 (p-p65) levels were reduced in PHLDA1 KO cells following X-ray treatment (**Figure 9B**). To confirm the similar role of PHLDA1 *in vivo*, we performed RNA *in situ* hybridization (ISH) for PHLDA1, p65, and COL1A1 in human kidney tissues. This analysis confirmed that PHLDA1 is expressed in proximal tubules and revealed a positive correlation between PHLDA1 upregulation and NF-κB activation in CKD (**Supplementary Figure S5**). Mechanistically, forced over-expression (OE) of p65 in PHLDA1-KO CD10^+^ cells rescued the expression of cytokines, including NFKBIA, IL-6, and IL-1β (**Figure 9C**). In summary, these results indicate that PHLDA1 modulates the inflammatory response of CD10^+^ PTE cells via the NF-κB pathway during renal fibrosis.

**Figure 10:**
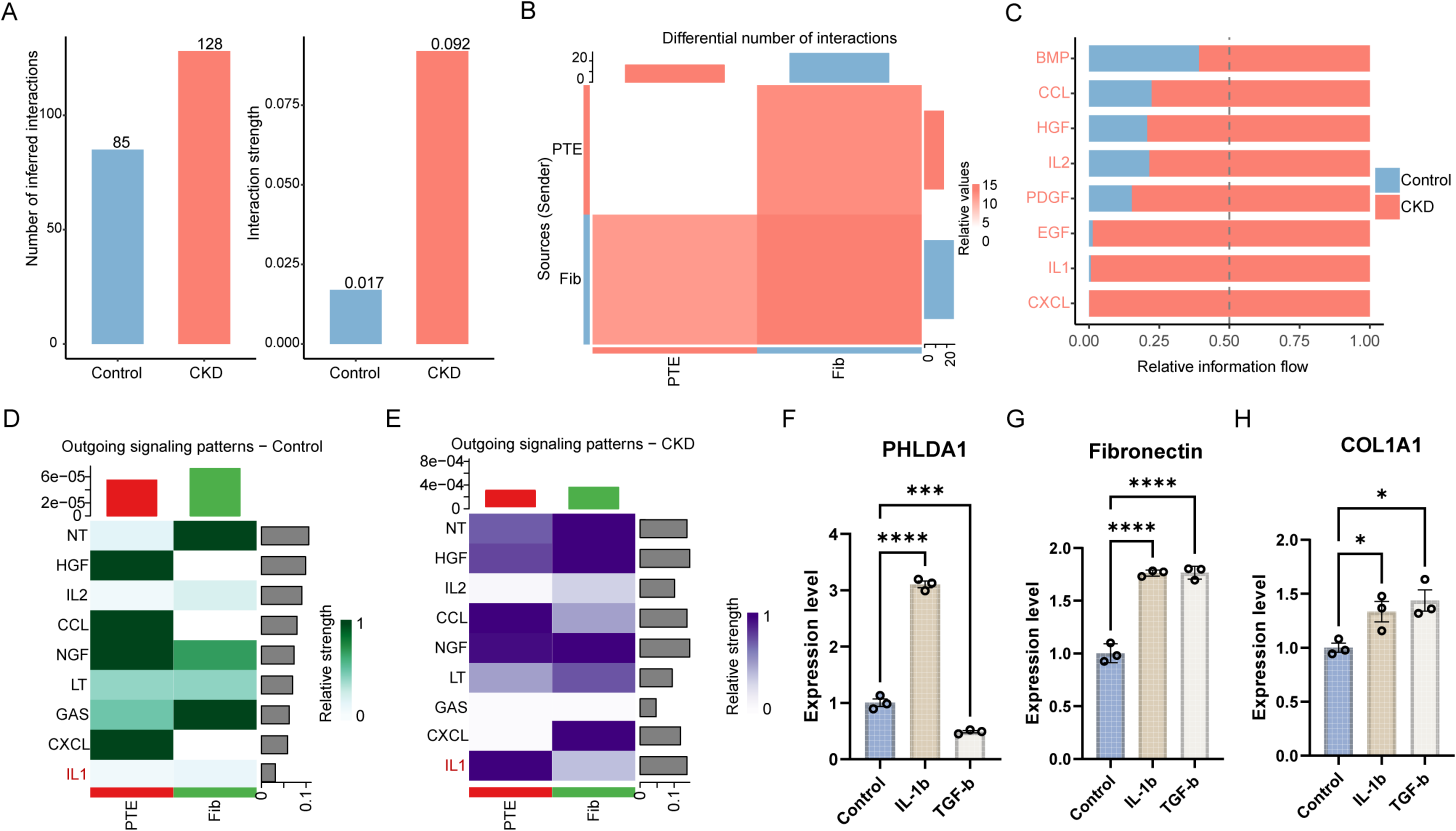
Enhanced PTE-fibroblast communication in renal fibrosis via IL-1 signaling. **(A)** Global CellChat summary of inferred intercellular communication between proximal tubular epithelial cells (PTEs) and fibroblasts in control versus CKD kidney, shown as the total number of inferred ligand–receptor interactions (left) and overall interaction strength (right). **(B)** Differential interaction heatmap comparing the number of interactions between the two major cell types (PTEs and fibroblasts) in CKD versus control, with sender (source) and receiver (target) cell types indicated. **(C)** Comparison of relative information flow for selected signalling pathways between control and CKD kidneys. **(D, E)** Heatmaps of outgoing signalling patterns from PTEs and fibroblasts under control **(D)** and CKD **(E)** conditions, highlighting changes in pathway-level communication, including IL-1 signalling.**(A, B)** Bar plots quantifying the number **(A)** and strength **(B)** of interactions between PTEs and fibroblasts in fibrotic versus control kidney. **(C)** Heatmap comparing information flow of selected signalling pathways between fibrotic and control kidneys. **(D, E)** Heatmaps depicting outgoing IL-1 signalling patterns from PTEs **(D)** and fibroblasts **(E)** in control and fibrotic conditions. **(F–H)** Bar graph showing the expression of PHLDA1 **(F)**, Fibronectin **(G)**, and COL1A1 **(H)** in PDGFRβ+ renal cells treated with 100ng/ml IL-1β or 15 ng/ml TGF-β. Ordinary one-way ANOVA tests were performed, and gene expression was normalized to GAPDH in F, G, and **(H)** Data are presented with mean ± SD; *p < 0.05, ****p < 0.0001. CKD, chronic kidney disease; PTE, proximal tubular epithelial cell(s); Fib, fibroblasts; BMP, bone morphogenetic protein; CCL, C–C motif chemokine ligand; HGF, hepatocyte growth factor; IL2, interleukin-2; PDGF, platelet-derived growth factor; EGF, epidermal growth factor; IL-1, interleukin-1; CXCL, C–X–C motif chemokine ligand; NGF, nerve growth factor; LT, lymphotoxin; PHLDA1, pleckstrin homology-like domain family A member 1; COL1A1, collagen type I alpha 1 chain; PDGFRβ, platelet-derived growth factor receptor beta; IL-1β, interleukin-1 beta; TGF-β, transforming growth factor beta; GAPDH, glyceraldehyde-3-phosphate dehydrogenase; qPCR, quantitative polymerase chain reaction.

To further understand the mechanisms of PTE and fibroblasts’ crosstalk, we performed cell-cell communication analysis based on single-nuclei sequencing data. Both the interaction number and strength between PTEs and fibroblasts were elevated in the fibrotic kidney group (**Figures 10A, B**). Besides the IL-1 signalling pathway, inflammatory (CXCL, IL2, CCL) and remodelling (BMP, HGF, PDGF, EGF) signalling pathways also had higher information flow in the fibrotic kidney samples (**Figure 10C**). Subsequent comparisons revealed that the IL-1 pathway was specifically activated in PTEs from the fibrotic group, but not in the control group (**Figures 10D, E**). Moreover, consistent with the experimental results in cardiac fibroblasts, PHLDA1 expression was significantly increased following stimulation with IL-1β, but was reduced in PDGFRβ^+^ kidney cells treated with TGF-β (**Figures 10F–H**). These data suggested that IL-1β mediated cell-cell communications between PTEs and fibroblasts may contribute to renal fibrosis.

## 4 Discussion

Fibrotic processes are key hallmarks of both cardiac dysfunction and CKD progression, two tightly integrated processes (6) (24) (25). However, the shared inflammation-induced fibrotic targets and mechanisms between the two organs were limited. In this study, we conducted a comprehensive analysis of public datasets from fibrotic heart and kidney tissues, integrating bulk RNA, snRNA, and ST-RNA sequencing layers. We demonstrate that *PHLDA1* is highly expressed in M1 macrophages in cardiac fibrosis and in proximal tubular epithelial cells in renal fibrosis, where it promotes inflammatory responses through the NF-κB pathway. Furthermore, cell-cell communication analysis and *in vitro* experiments reveal that IL-1β stimulation enhances PHLDA1 expression and mediates intercellular crosstalk, contributing to fibrotic progression. These findings highlight the organ-specific yet overlapping molecular mechanisms of inflammatory fibrosis mediated by IL-1β–PHLDA1 signalling pathway. Given the difficulties of developing a cardio-renal comorbidity animal model, further investigation of the IL-1β–PHLDA1 axis in individual disease models may provide more information about developing therapeutic strategies targeting PHLDA1. Our bioinformatic analyses and functional identified IL-10 and PHLDA1 as central targets implicated in both cardiac and renal fibrosis from a diagnostic perspective.

The Notch signaling pathway mediated the reduction of oxidative stress, prevented cardiomyocyte apoptosis, and alleviated fibrosis (26). In the present study, GSEA and GSVA analyses revealed that the Notch signaling pathway was the most significant hallmark in HF samples but not CKD samples, where the G2M checkpoint hallmark emerged as the most upregulated pathway in CKD datasets (**Supplementary Figure S6**) (27). Moreover, we found that both interferon-γ and interferon-α signaling pathways were significantly activated in the HF and CKD. This observation is consistent with previous studies and suggests that chronic or dysregulated viral infection may contribute to the development of cardiac and renal fibrosis (28, 29).

GO analysis of shared genes revealed impaired degradation of proteins and peptides, which may accelerate extracellular matrix (ECM) accumulation in both diseases. Moreover, IL-12-related biological processes emerged as key features in the GO enrichment results (**Figure 3A**). These findings are supported by recent studies showing that IL-12β reduces inflammation by regulating T cells and macrophages in heart failure, and that IL-12β has been identified as a significant cytokine in IgA nephropathy (30, 31). Taken together, this cross-organ perspective analysis highlights IL-12β as a potential therapeutic target for cardiac and renal comorbidly.

Except for inflammation-related pathways, aldosterone and foxO signalling were found to be significant mediators (32–35). Interestingly, FoxO1 is reported to be promoted by PHLDA1 during inflammatory responses (36).

We also unveiled several diagnostic genes by random forest selection for cardiac and renal fibrosis. ‘Interleukin 1 Receptor Like 1’ (IL1RL1), has been reported to be involved in sepsis, obesity, asthma, and several other diseases by regulating the inflammation process (37). Soluble IL1RL1 in serum has been recently proven able to predict the risk of cardiac remodelling of participants with CKD (38). In this study, we found that IL1RL1 was the most significant diagnostic gene (AUC = 0.971, **Supplementary Figure S7A**) among all the genes, not only at the protein level in both diseases. Another identified diagnostic gene for both diseases in our study (AUC = 0.963, **Supplementary Figure S7B**), ‘Tolloid-like 2’ (TLL2 or NETO2), was previously reported in carcinoma progression by increasing cell migration and regulating immune filtration (39, 40). However, this gene has never been reported to be associated with cardiac or renal inflammation and fibrosis, though its biological process was predicted as collagen fibril organization (GO term: 0030199).

IL-10 is a crucial anti-inflammatory cytokine involved in regulating immune responses and protecting tissues from excessive damage in conditions like HF (41) and CKD (42). It plays a protective role by suppressing inflammation. In our current study, we found that the tissue-level expression of IL-10 was consistently reduced in both HF and CKD samples, especially at the end stage of the diseases (**Figures 3G, I, L**). Therefore, such kind of expression pattern could be considered as a diagnostic marker, where low expression levels of IL-10 indicate tissue fibrosis. This reduction in expression may reflect the exhaustion of the compensatory anti-inflammatory response as the disease progresses (43), which was consistent with the previous clinical study that IL-10 was decreased in HF with reduced ejection fraction (44) and non-dialysis end-stage CKD patients (45). Despite this reduced expression, IL-10 continues to exert protective effects at the end stage, although its overall protective function is attenuated. This attenuation in expression may contribute to the progression of fibrosis, as indicated by IL-10’s central role in the protein-protein interaction (PPI) network analysis (**Figure 3B**). However, whether the diagnostic ability or protective effect of IL-10 varies across different stages of cardiac and renal fibrosis remains unresolved in our current study, as we did not include sequencing samples from various stages of the diseases.

PHLDA1 has been repeatedly implicated in immune infiltration across several diseases, including heart failure, cardiomyopathy, and CKD (46) (47). Nevertheless, these studies did not clarify how PHLDA1 contributes to cardiac fibrosis. Here, we clarified that PHLDA1 was increased in the M1 macrophage in fibrotic heart from snRNA level and co-expressed with IL-10 in M1 macrophages. Moreover, regarding fibrotic kidney, our results demonstrated that PHLDA1 regulated the inflammation level of PTE cells and communicated with PDGFRβ^+^ cells via IL-1β. Taken together, our data uncovered that PHLDA1 was both diagnostic and functional gene in cardiac and renal fibrosis.

Within the regulatory network of M1 macrophages in the fibrotic heart, MAFF emerged as a shared transcription factor (TF) for both PHLDA1 and IL-10, although the regulatory directions were opposite (**Figure 5D**). While direct evidence linking MAFF to M1 macrophages in cardiac or renal fibrosis remains limited, recent studies have identified MAFF as a key TF driving pro-inflammatory cytokine production in neutrophils stimulated by LPS and as a regulator of inflammatory responses in atherosclerosis (48, 49). However, since the low detected macrophage number in the control group (n = 186) precluded reliable sub−clustering of macrophages into M1 and M2 subsets, it restricted the investigation of the significance of IL-10 and MAFF in renal fibrosis using the fibrotic kidney snRNA-seq dataset (GSE195718). As IL−10 and MAFF are predominantly expressed within macrophage subpopulations, further study that harbors a larger sample size of the sequencing are required to delineate their roles in CKD (**Supplementary Figure S4**).

TGF-β and IL-1β have both been reported to activate cardiac and renal fibrosis (6, 50). Our results revealed that IL-1β significantly mediated cell-cell crosstalk during cardiac and renal fibrosis. Accordingly, TGF-β and IL-1β were used to stimulate cardiac fibroblasts and PDGFRβ^+^ kidney cells. Intriguingly, PHLDA1 expression was suppressed following TGF-β stimulation but increased upon IL-1β stimulation (**Figures 7F**, **9F**). One possible explanation is that IL-1β reprograms fibroblasts toward an immune-like phenotype in which PHLDA1 is activated, whereas TGF-β drives fibroblasts toward a matrix-producing phenotype in which PHLDA1 is suppressed (51–53).

Our study also suffers from several limitations. Firstly, the sample size of the sequencing data is relatively small. Therefore, a larger, well-phenotyped cohort should be recruited for our further studies. Secondly, due to the limited diversity of the disease stages among the sequencing samples, we could not exclude the possibility that the diagnostic performance of the genes can vary within different stages of cardiac or renal fibrosis. Hence, the diagnostic ability of the genes should be tested in the cohort with stage-wise information of the diseases before any clinical application.

In summary, we provide first comprehensive multi-omics study of cardiac and renal fibrosis comorbidity and identified that IL-1β mediated cell-cell communications between PTEs and fibroblasts may contribute to renal fibrosis.

## Data Availability

The datasets presented in this study can be found in online repositories. The names of the repository/repositories and accession number(s) can be found below: GSE313814 (GEO).

## Supplementary material

Supplementary Figure 1 Fluorescence-Activated Cell Sorting (FACS) for primary CD10 + PTE cells from human kidney.

Supplementary Figure 2 Flow cytometry analysis of transfecting efficiency in PHLDA1-KO-CD10^+^ PTE cells.

Supplementary Figure 3 Supplemental information of the key gene selection for cardiac and renal fibrosis. **(A, B)** Volcano plot of differentially expressed genes in GSE141910 **(A)** and GSE66494 **(B)**. **(C, D)** Coefficient plot **(C)** and binomial deviance plot **(D)** during the LASSO regression and cross-validation for the selection of the key genes in cardiac and renal fibrosis. **(E)** Receiver operating characteristic (ROC) curves evaluating the diagnostic performance of IL10 and PHLDA1 for discriminating disease samples from normal controls in the independent renal validation dataset GSE104948, with corresponding AUC values. **(G, H)** Violin plots showing normalized expression of IL10 **(G)** and PHLDA1 **(H)** in GSE104948 (Normal vs ANCA-associated vasculitis). Statistical significance determined by the two-sided t-test; **p < 0.05*.

Supplementary Figure 4 Key cell types annotation in single-nuclei kidney dataset GSE195718.

Supplementary Figure 5 Representative image of combined immunofluorescent and multiplex RNA ISH of PHLDA1 and p65. Scale bars, 50 µm.

Supplementary Figure 6 GSVA analysis of fibrotic kidney datasets GSE66494.

Supplementary Figure 7 ROC curve of IL1RL1 and TLL2 gene in merged fibrotic heart and kidney datasets.
