# Supplementary figures and images for: PHLDA1 is a shared diagnostic and key mediator of inflammatory fibrosis in heart and kidney
Source: Front Immunol. 2026 Feb 5;17:1765221. doi: 10.3389/fimmu.2026.1765221 (PMC12917609; doi:10.3389/fimmu.2026.1765221)

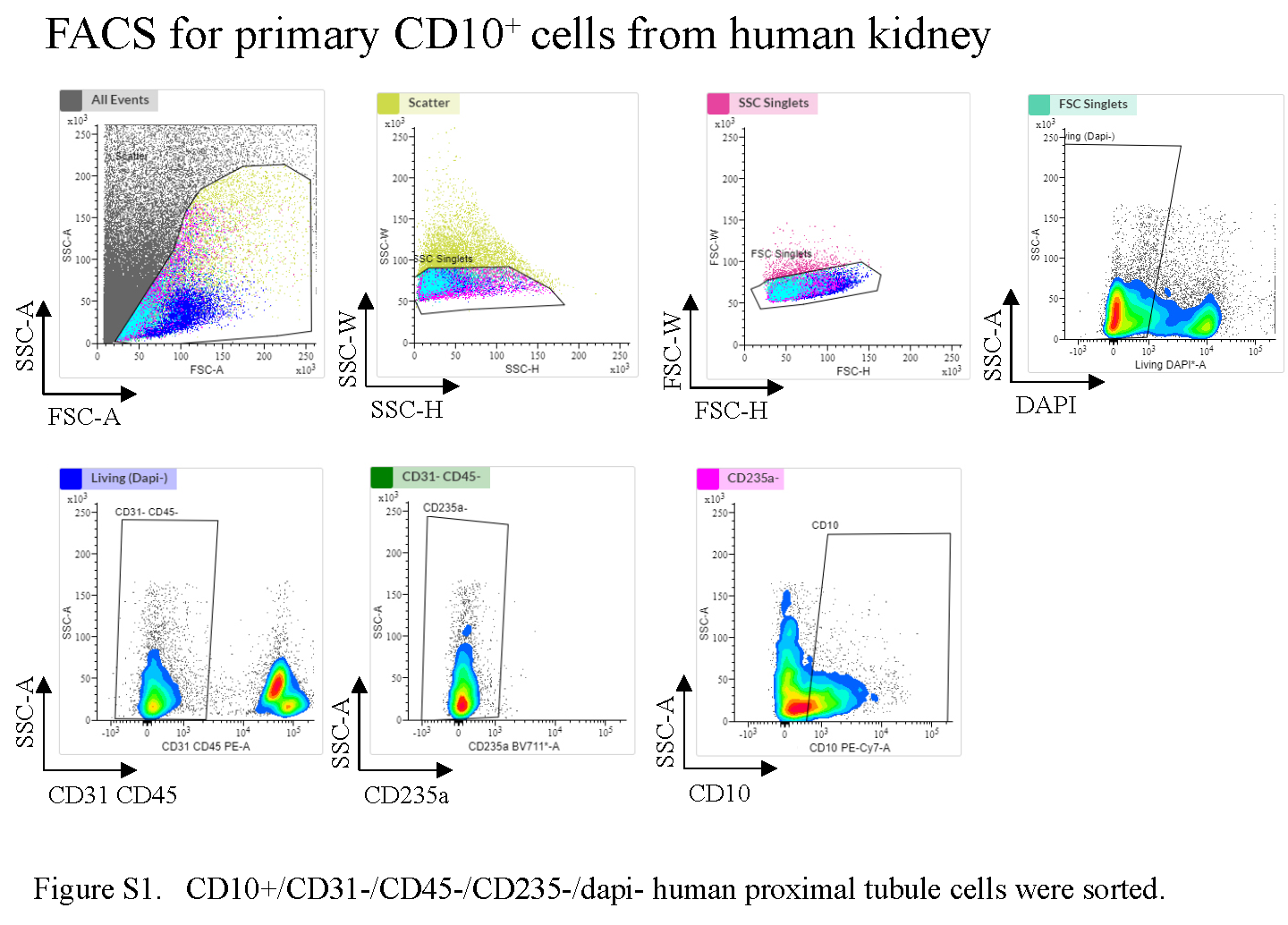

Supplement: Supplementary Figure 1 — Fluorescence-Activated Cell Sorting (FACS) for primary CD10 + PTE cells from human kidney. [file Image1.tiff]

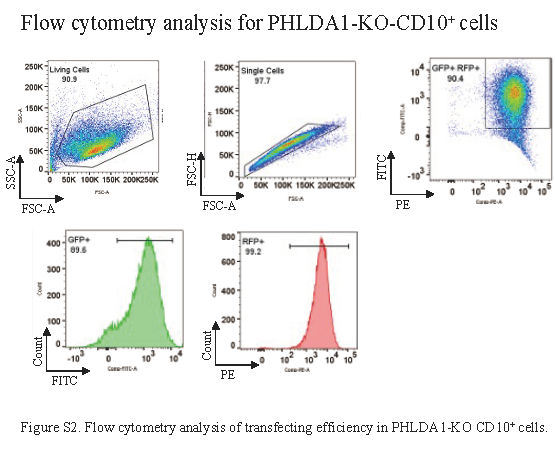

Supplement: Supplementary Figure 2 — Flow cytometry analysis of transfecting efficiency in PHLDA1-KO-CD10+ PTE cells. [file Image2.tiff]

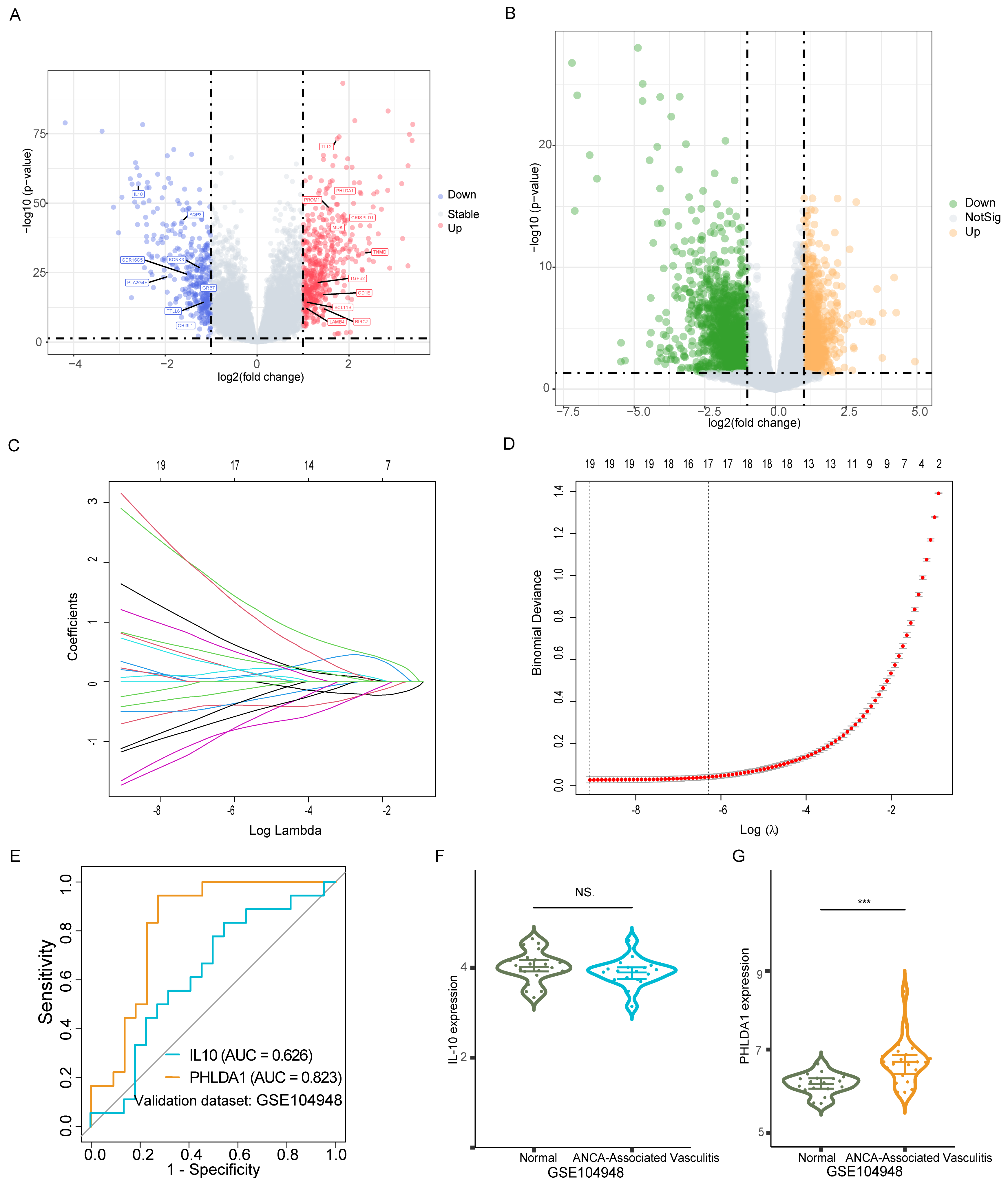

Supplement: Supplementary Figure 3 — Supplemental information of the key gene selection for cardiac and renal fibrosis. (A, B) Volcano plot of differentially expressed genes in GSE141910 (A) and GSE66494 (B). (C, D) Coefficient plot (C) and binomial deviance plot (D) during the LASSO regression and cross-validation for the selection of the key genes in cardiac and renal fibrosis. (E) Receiver operating characteristic (ROC) curves evaluating the diagnostic performance of IL10 and PHLDA1 for discriminating disease samples from normal controls in the independent renal validation dataset GSE104948, with corresponding AUC values. (G, H) Violin plots showing normalized expression of IL10 (G) and PHLDA1 (H) in GSE104948 (Normal vs ANCA-associated vasculitis). Statistical significance determined by the two-sided t-test; *p < 0.05. [file Image3.tif]

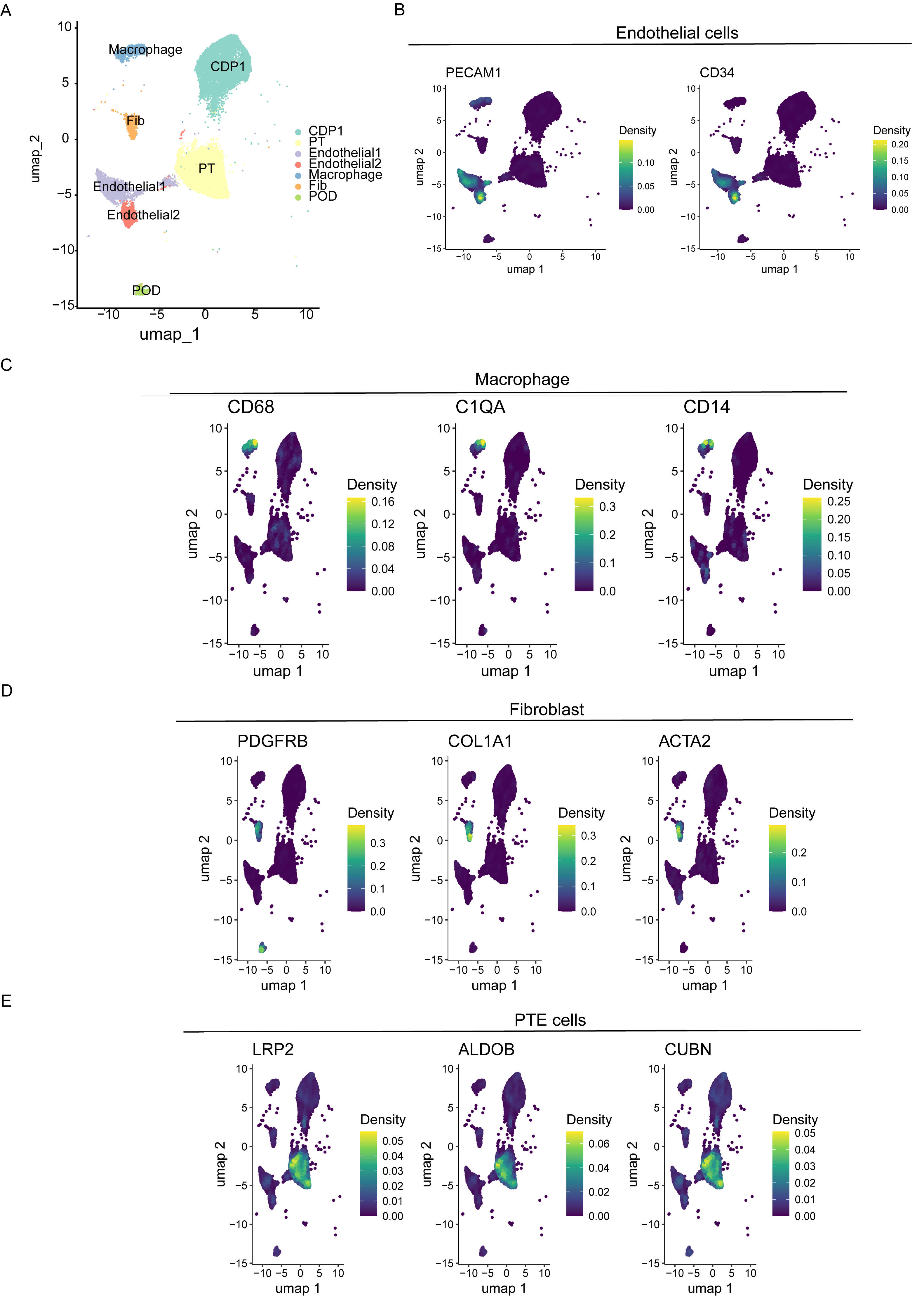

Supplement: Supplementary Figure 4 — Key cell types annotation in single-nuclei kidney dataset GSE195718. [file Image4.tif]

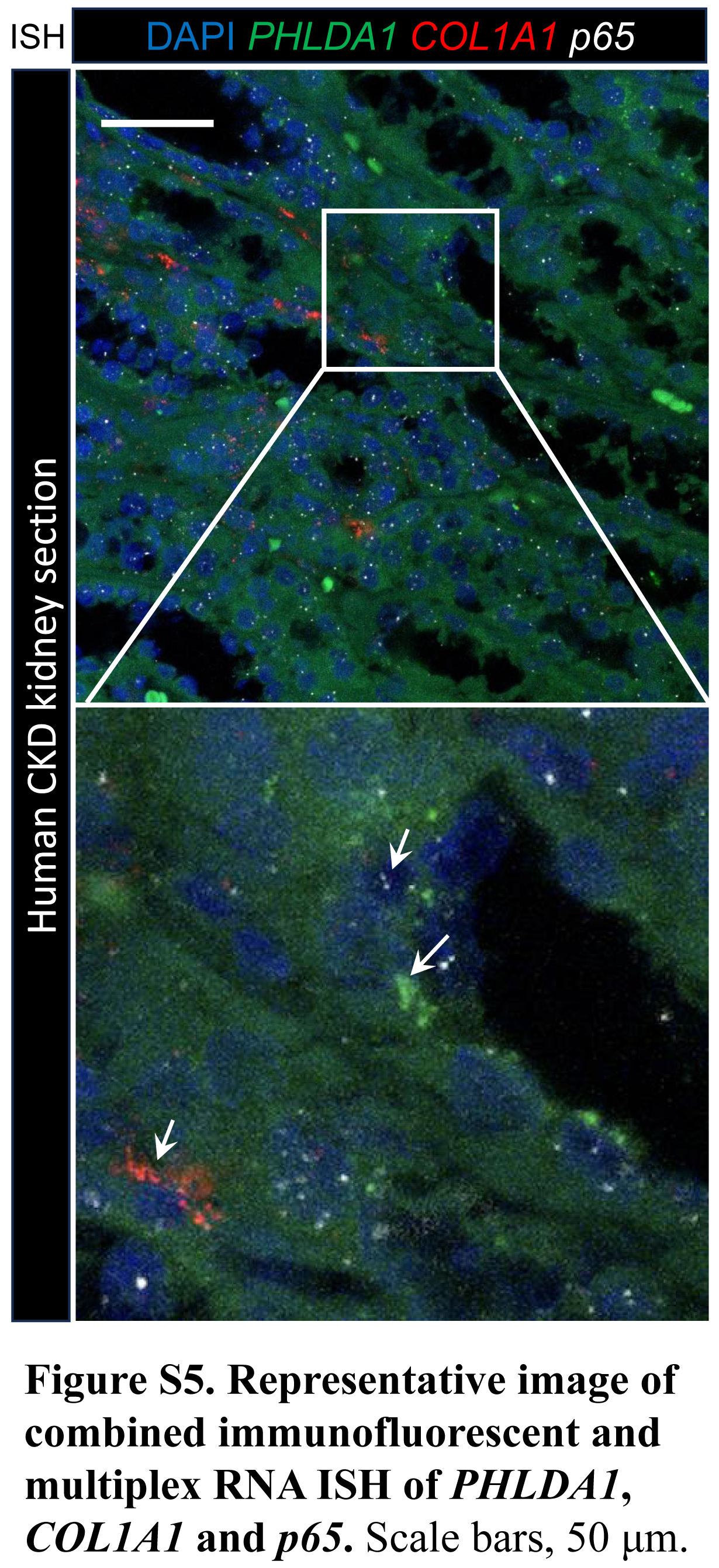

Supplement: Supplementary Figure 5 — Representative image of combined immunofluorescent and multiplex RNA ISH of PHLDA1 and p65. Scale bars, 50 µm. [file Image5.tif]

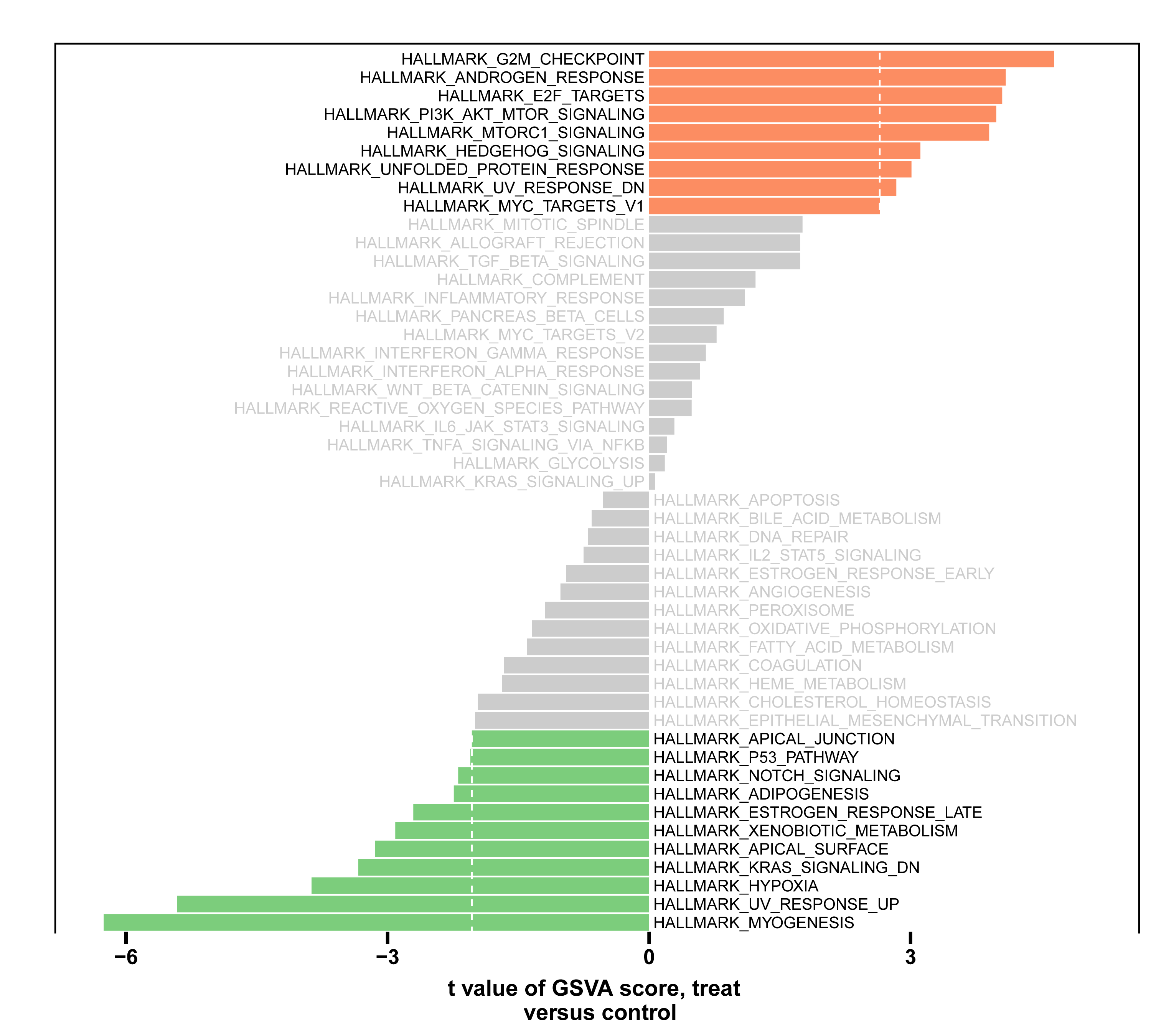

Supplement: Supplementary Figure 6 — GSVA analysis of fibrotic kidney datasets GSE66494. [file Image6.png]

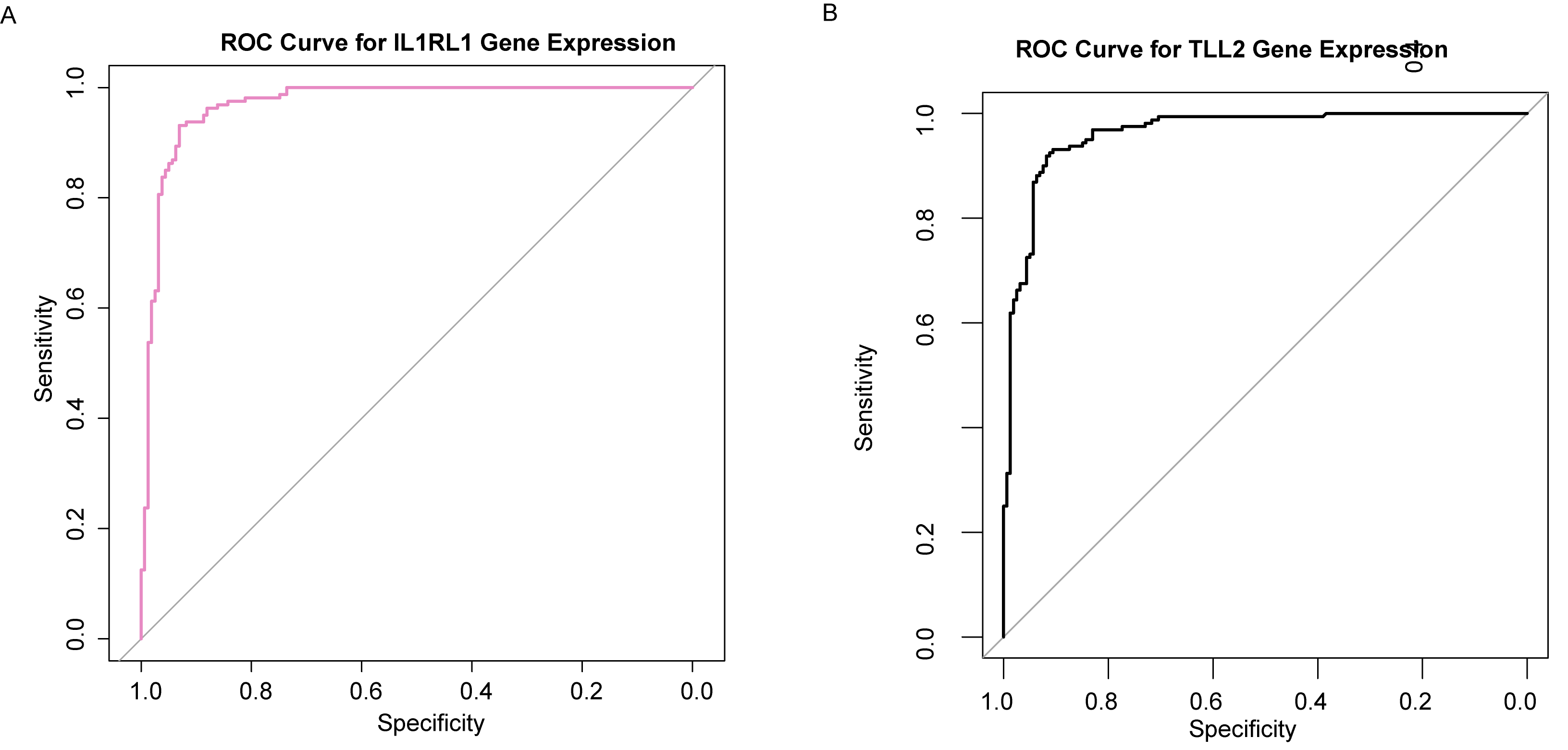

Supplement: Supplementary Figure 7 — ROC curve of IL1RL1 and TLL2 gene in merged fibrotic heart and kidney datasets. [file Image7.png]
