# Supplementary material for: PHLDA1 is a shared diagnostic and key mediator of inflammatory fibrosis in heart and kidney
Source: Front Immunol. 2026 Feb 5;17:1765221. doi: 10.3389/fimmu.2026.1765221 (PMC12917609; doi:10.3389/fimmu.2026.1765221)
Supplement: Supplementary file 10 [file Table1.docx]

**Table S1. Primer sequences used in this study.**

| Genes | Organism | Forward Primers (5'-3') | Reverse Primers (5'-3') |
| --- | --- | --- | --- |
| PHLDA1 | Human | TCAACCGAAAGGGCAGATCC | CAGTGAGGCAAGAGACAGCA |
| MAFF | Human | CCACAACAAAACTCAGCGCA | GGGTCATTCGGTCCCAGTTT |
| IL-1β | Human | ACCGAGGCTTATGTGCACAT | AGACATCACCAAGCTTTTTTGCT |
| IL-6 | Human | CCTTCTCCACAAACATGTAACAAGA | TCACCAGGCAAGTCTCCTCA |
| COL1A1 | Human | AAAGATGGACTCAACGGTCTC | CTTCCAGTCAGAGTGGCACATCTTGAG |
| GAPDH | Human | GAAGGTGAAGGTCGGAGTCA | TGGACTCCACGACGTACTCA |
| Fibronectin | Human | AACAAACACTAATGTTAATTGCCCA | TCGGGAATCTTCTCTGTCAGC |
| Phlda1 | Mice | GTGATGACGGAGGGCAAAGA | CCCTCTTCAGGCAGAGTTGG |
| gapdh | Mice | GGTGAAGGTCGGTGTGAAC | GAGTCAATGAAGGGGTCGTTG |
