# Supplementary material for: PHLDA1 is a shared diagnostic and key mediator of inflammatory fibrosis in heart and kidney
Source: Front Immunol. 2026 Feb 5;17:1765221. doi: 10.3389/fimmu.2026.1765221 (PMC12917609; doi:10.3389/fimmu.2026.1765221)
Supplement: Supplementary file 11 [file Table2.docx]

**Table S2. Genes selected by LASSO.**

| Gene symbol | Full name |
| --- | --- |
| IL10 | Interleukin 10 |
| TLL2 | Tolloid-like 2 |
| PHLDA1 | Pleckstrin Homology-Like Domain Family A Member 1 |
| PROM1 | Prominin 1 |
| AQP3 | Aquaporin 3 |
| CRISPLD1 | Cysteine-Rich Secretory Protein LCCL Domain Containing 1 |
| MDK | Midkine |
| TNMD | Tenomodulin |
| PLA2G4F | Phospholipase A2 Group IV F |
| KCNK3 | Potassium Channel Subfamily K Member 3 |
| GRB7 | Growth Factor Receptor-Bound Protein 7 |
| BIRC7 | Baculoviral IAP Repeat-Containing 7 |
| SDR16C5 | Short Chain Dehydrogenase/Reductase Family 16C Member 5 |
| TGFB2 | Transforming Growth Factor Beta 2 |
| CD1E | CD1E Molecule |
| BCL11B | B-Cell CLL/Lymphoma 11B |
| CHI3L1 | Chitinase 3-Like 1 |
| TTLL6 | Tubulin Tyrosine Ligase-Like 6 |
| LAMB4 | Laminin Subunit Beta 4 |
