# Supplementary material for: PHLDA1 is a shared diagnostic and key mediator of inflammatory fibrosis in heart and kidney
Source: Front Immunol. 2026 Feb 5;17:1765221. doi: 10.3389/fimmu.2026.1765221 (PMC12917609; doi:10.3389/fimmu.2026.1765221)
Supplement: Supplementary file 12 [file Table3.docx]

Table S3. Detailed information of the validation dataset for the diagnostic ability of IL-10 and PHLDA1.

| ACCESSION  NUMBER | Total sample size | Data type | Tissue | Control sample size | Condition |
| --- | --- | --- | --- | --- | --- |
| GSE57338 | 218 | Bulk RNA | Left Ventricle | 136 | Normal VS Heart Failure |
| GSE66494 | 40 | Bulk RNA | Renal biopsy | 18 | Normal VS ANCA-Associated Vasculitis |
