# Supplementary material for: PHLDA1 is a shared diagnostic and key mediator of inflammatory fibrosis in heart and kidney
Source: Front Immunol. 2026 Feb 5;17:1765221. doi: 10.3389/fimmu.2026.1765221 (PMC12917609; doi:10.3389/fimmu.2026.1765221)
Supplement: Supplementary file 13 [file Table4.docx]

Table S4. Bioinformatic analysis identified shared key genes, pathways, and biological processes between cardiac and renal inflammatory fibrosis.

| Type | Items |
| --- | --- |
| Key genes | Interleukin 10 (IL-10) |
|  | Pleckstrin Homology-Like Domain Family A Member 1 (PHLDA1) |
|  | Tolloid-like 2 (TLL2) |
|  | Interleukin 1 Receptor Like 1 (IL1RL1) |
| Pathways | TNFα signaling via NF-κB |
|  | Interferon-α / Interferon-γ responses |
|  | Cytokine–cytokine receptor interaction |
|  | Osteoclast differentiation |
|  | Aldosterone secretion signalling |
|  | Complement cascades signalling |
|  | Osteoblast differentiation signalling |
|  | Rein-angiotensin system |
|  | FoxO signalling pathway |
| Biological processes | Response to wounding |
|  | IL-12 production and regulation |
|  | Negative regulation of proteolysis |
|  | Apoptotic process |
|  | Neutrophil chemotaxis |
